# Rac1 Suppression by the Focal Adhesion Protein GIT ArfGAP2 and Podocyte Protection

**DOI:** 10.1681/ASN.0000000614

**Published:** 2025-02-28

**Authors:** Naoyuki Shimada, Jun Matsuda, Kana Asano-Matsuda, Maho Tokuchi, Lamine Aoudjit, Agnieszka Masztalerz, Serge Lemay, Tomoko Takano, Yoshitaka Isaka

**Affiliations:** 1 Department of Nephrology, Graduate School of Medicine, The University of Osaka, Suita, Osaka, Japan; 2 Division of Nephrology, McGill University Health Centre, Montreal, Quebec, Canada

**Keywords:** cell biology and structure, glomerular disease, glomerular epithelial cells, podocyte

## Abstract

**Key Points:**

- Focal adhesion protein GIT2 protected podocytes from injury in rodent proteinuric disease models.
- GIT2 facilitated translocation of tyrosine phosphatase PTP1B to focal adhesions where it dephosphorylates p130Cas, thereby suppressing Rac1 activity.
- Stabilizing GIT2 or facilitating GIT2 localization to focal adhesions in podocytes could be a therapeutic strategy in proteinuric kidney diseases.

**Background:**

Podocytes have an intricate structure featured by numerous actin-based projections called foot processes. Rho family of small GTPases, including Ras-related C3 botulinum toxin substrate 1 (Rac1), play important roles in actin cytoskeletal remodeling required for cell morphology and adhesion. We previously showed that Rac1 activation in podocytes causes foot process effacement and proteinuria, but the upstream and spatiotemporal regulatory mechanism directing Rac1 is largely unknown. Recently, we identified the focal adhesion protein GIT ArfGAP2 (GIT2) as one of the Rac1 interactors in human podocytes by proximity-dependent biotin identification and proteomics.

**Methods:**

Systemic and podocyte-specific GIT2 knockout mice were generated and assessed for kidney phenotypes. Human podocytes with GIT2 knockdown (KD) and overexpression were established using lentiviral transduction and characterized.

**Results:**

GIT2 was enriched in glomeruli, including podocytes, in the mouse kidney. Gene deletion of *Git2* in podocytes caused exacerbated proteinuria and foot process effacement when subjected to the minimal change disease model and salt-sensitive hypertension model, which were improved by pharmacological inhibition of Rac1. In cultured podocytes, GIT2 KD resulted in Rac1-dependent cell spreading with marked lamellipodial protrusions, accelerated focal adhesion disassembly, and shorter focal adhesion lifetime. In GIT2 KD podocytes, tyrosine phosphorylation of the focal adhesion protein p130 Crk-associated substrate (Cas) was significantly increased, accompanied by impaired localization of the tyrosine phosphatase PTP1B to focal adhesions. These phenotypes observed in GIT2 KD podocytes were reversed by GIT2 overexpression.

**Conclusions:**

The results indicate that GIT2 facilitates translocation of PTP1B to focal adhesions where it dephosphorylates p130Cas, thereby suppressing local Rac1 activity and protecting against podocyte injury and proteinuria.

## Visual Abstract

**Figure:**
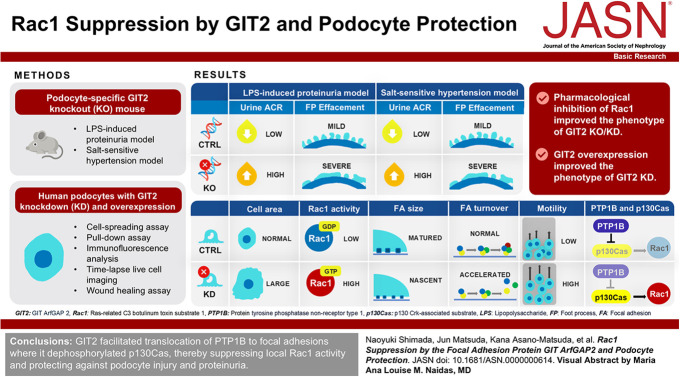

## Introduction

Podocytes have an intricate structure characterized by primary, secondary, and tertiary projections termed foot processes.^1^ The interdigitating finger-like foot processes of adjacent podocytes form a gap called the slit diaphragm. Podocytes attach to the outer aspects of the glomerular basement membrane through the sole of the foot process by focal adhesions. The unique morphology of the foot process is supported by their well-organized actin cytoskeleton and is critical for podocyte function.^2–4^ The common morphological change of injured podocytes is foot process effacement, which is attributable to the rearrangement of the actin cytoskeleton.

The Rho family of small GTPases (Rho GTPases) is the master regulator of the actin cytoskeletal dynamics. Rho GTPases are molecular switches that shuttle between active (GTP-bound) and inactive (GDP-bound) forms. In the active state, they interact with a large number of effectors, which regulate biological processes such as cell morphology, motility, and adhesion.^5,6^ The cycling of Rho GTPases is regulated by three families of proteins: guanine nucleotide exchange factors, GTPase-activating proteins, and GDP dissociation inhibitors.^5,6^

Of the 20 members of Rho GTPases, Ras-related C3 botulinum toxin substrate 1 (Rac1) is one of the prototypical and best characterized Rho GTPases in podocytes.^4,7,8^ The importance of the tight regulatory control of Rac1 activity for podocyte function is well supported by previous studies. We and others reported that hyperactivation of Rac1 in murine podocytes causes foot process effacement and proteinuria.^9,10^ Activated Rac1 has been demonstrated in podocytes from patients with minimal change disease and FSGS^10^ and from rodents with salt-sensitive hypertension.^11,12^ Genetic analyses have shown that loss-of-function mutations of one of the GDP dissociation inhibitors, *ARHGDIA*, cause congenital or child-onset FSGS due to Rac1 activation.^13–15^

To achieve a better understanding of the regulatory mechanism of Rac1, we recently reported proximity-dependent biotin identification proteomics using Rac1 as bait in cultured human podocytes.^16^ We identified GIT ArfGAP2 (GIT2) as an interacting protein with Rac1 in podocytes. GIT2 is nearly ubiquitously expressed in mammalian tissues.^17^ GIT2 function has been studied using mice with systemic *Git2* deletion. Mazaki *et al.* demonstrated that GIT2 plays an important role for directional chemotaxis in neutrophils,^18^ which was the first report concerning the role of GIT2 *in vivo*. Afterward, several groups have suggested that GIT2 is necessary for maturation/proliferation and chemokine-mediated motility in thymocytes.^19,20^ In another report, Git2-deficient mice displayed anxiety-like behavior,^21^ suggesting possible neurobehavioral function of GIT2. In more recent studies, Git2-deficient mice were protected against obesity under high-fat diet because of catabolic shift from glucose to lipid likely by aberrant hypothalamic activity^22^ but showed glucose intolerance due to disruption of pancreatic *β*-cell development,^23^ suggesting a possible role of GIT2 toward the control of somatic energy metabolism. Despite its wide distribution and suggested multifunction,^22^ neither the gene expression nor functional roles of GIT2 in the kidney, including podocytes, have been investigated. In this study, we aim at studying the expression and role of GIT2 in the kidney.

## Methods

### Experimental Animals

Mice were of C57BL/6J background. All procedures were approved by the Animal Experiment Committee of The University of Osaka and were in accordance with relevant guidelines. Systemic *Git2* knockout (GIT2^DEL/DEL^) and *Git2* floxed mice were established by CLICK (CRISPR with long single-stranded DNA inducing conditional knockout alleles) as previously reported.^24^ *Nphs2 Cre* mice have been described previously.^25,26^ *Git2* floxed mice were crossed with *Nphs2 Cre* mice to generate podocyte-specific *Git2* knockout (GIT2^Pod−/−^) mice. Age-matched GIT2^+/+^ and GIT2^DEL/+^ mice were used as controls for GIT2^DEL/DEL^ mice and *Cre*-negative mice were for GIT2^Pod−/−^ mice.

### Cell Culture

Immortalized human podocytes (XimBio, CIHP-1) were cultured in RPMI1640 containing 10% FBS and 1% penicillin/streptomycin (Gibco) at permissive conditions (33°C in 5% CO_2_) and then differentiated under nonpermissive conditions (37°C in 5% CO_2_) for 5 days. Podocytes with GIT2 knockdown (KD) and overexpression were established using lentiviral transduction.

### Statistical Analyses

All results are expressed as mean±SEM. Statistical analyses were conducted using JMP software (SAS Institute) and Prism software (GraphPad). Focal adhesion lifetime was analyzed by Fisher's exact test. For others, multiple-group comparisons were performed using the Tukey–Kramer or Dunnett test. Differences between two experimental values were assessed by *t* test. *P* < 0.05 was considered statistically significant.

The detailed methodology is described in the Supplemental Methods.

## Results

### GIT2 Was Expressed in the Glomerulus, Including in Podocytes

We first studied the expression of GIT2 in the mouse kidney. Although GIT2 is known to be expressed ubiquitously, immunoblotting detected only a weak signal from the lysate of kidney cortex, as compared with the spleen (positive control) (Figure 1A). By contrast, the lysate of isolated glomeruli showed two distinct bands corresponding to those in the spleen (Figure 1A), suggesting that GIT2 expression is relatively enriched in the glomerulus in the kidney cortex. Localization of GIT2 protein by immunohistochemistry was not possible because none of the commercially available GIT2 antibodies showed specific staining when the *Git2*-deficient mouse kidney (below) was used as negative control. Thus, we used *in situ* hybridization to detect *Git2* mRNA using RNAscope. In low magnification, enriched *Git2* signals in glomerular tufts were appreciated (Figure 1B). In high magnification, *Git2* mRNA was expressed broadly in the glomerulus; some *Git2* mRNA was clearly expressed in WT1 transcription factor 1 (*Wt1*)-expressing podocytes, whereas the remainder was mostly in platelet-derived growth factor receptor (*Pdgfr*)-expressing mesangial cells and platelet and endothelial cell adhesion molecule 1 (*Pecam1*)-expressing endothelial cells (Figure 1C). This distribution of mRNA was consistent with a single-cell RNA sequencing data from the human kidney^27,28^ and indicates that GIT2 is expressed in podocytes and other glomerular cells.

Figure 1. Podocyte-specific *Git2*-deficient mice exhibit increased susceptibility to proteinuria.

**Figure:**
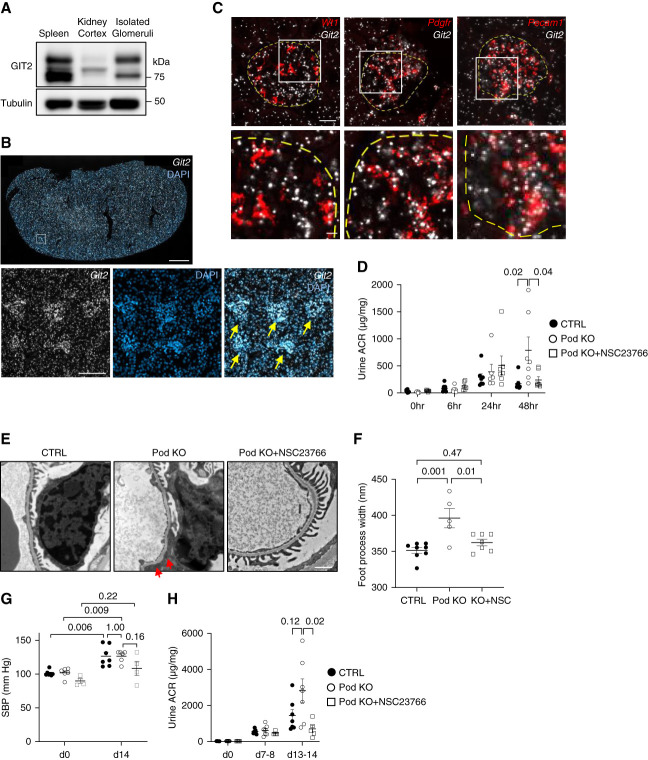
(A) Representative immunoblots for GIT2 and tubulin using lysates of mice kidney cortex and isolated glomeruli. Spleen lysate was used as a positive control. Equal amounts of protein (11 *µ*g) were separated by SDS PAGE. GIT2 expression (75 and 85 kDa) is relatively enriched in the glomerulus in mice kidney. Note that a faint band (just above 75 kDa) observed in the lane of kidney cortex is nonspecific. (B) Representative images of *in situ* hybridization for *Git2* mRNA in mice kidney. DAPI was used for nuclear counterstaining. Yellow arrows show glomerular tufts. (C) Comparison of *Git2* transcript in resident glomerular cell types: *Wt1*, *Pdgfr*, and *Pecam1* were used as a marker of podocytes, mesangial cells, and endothelial cells, respectively. Yellow dashed lines show contours of the glomeruli. (D–F) LPS-induced proteinuria model. (D) Quantification of urine ACR from control, podocyte-specific *Git2*-deficient (Pod KO) and NSC23766-treated Pod KO mice before and after 6, 24, and 48 hours of LPS injection. (E) Representative transmission electron microscopy of the glomeruli after 48 hours of LPS injection. (F) Quantification of foot process width in (E). (G–M) Salt-sensitive hypertension model. (G) Systolic BP was increased similarly at day 14 by uninephrectomy–DOCA/salt treatment in Pod KO and control mice while not significantly elevated in NSC23766-treated Pod KO mice. (H) Quantification of urine ACR before and at days 7–8 and days 13–14 after uninephrectomy–DOCA/salt treatment. (I) Representative transmission electron microscopy of the glomeruli at day 14 of uninephrectomy–DOCA/salt treatment. (J) Quantification of foot process width in (I).

**Figure:**
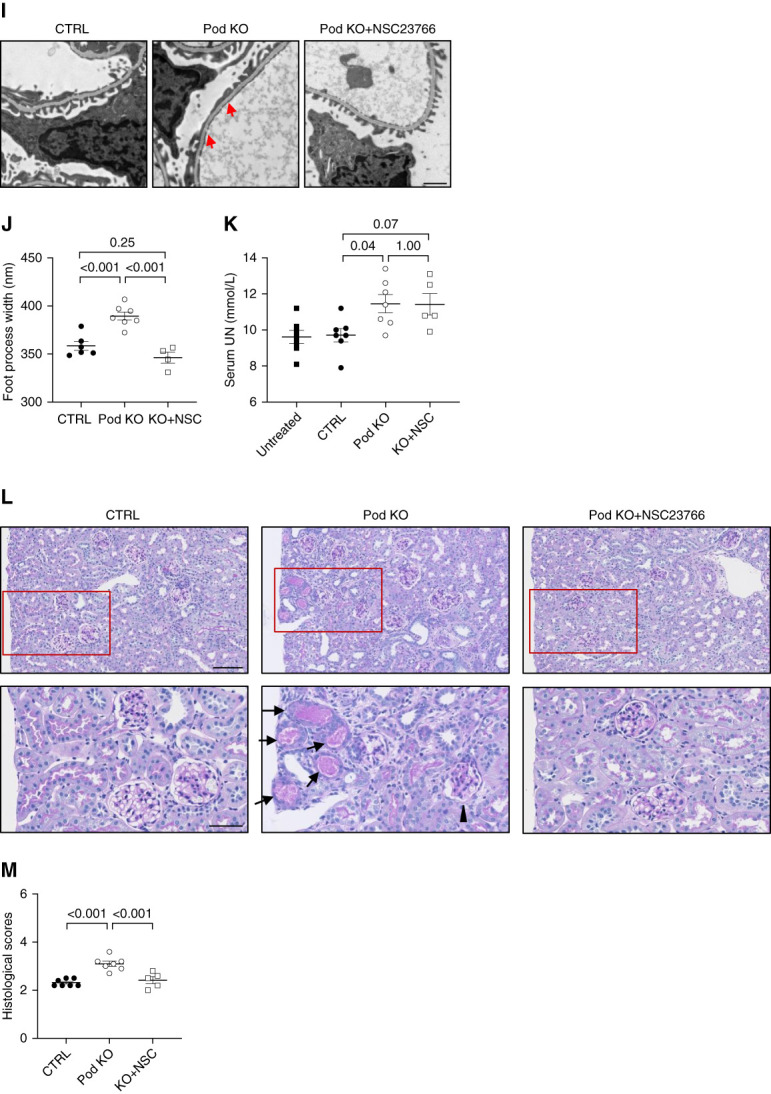
(K) Serum levels of UN at day 14 of uninephrectomy–DOCA/salt treatment. The levels from untreated mice are also shown as a reference. (L) Representative images of periodic acid–Schiff staining at day 14 of uninephrectomy–DOCA/salt treatment. Protein casts in atrophic tubules (arrows) and a collapsed glomerulus (arrowhead) in Pod KO mice are shown. (M) Quantification of tubulointerstitial damage in (L). Composite scores of leukocyte infiltration and tubular damage: 1=none/trace, 2=mild/patchy, and 3=severe/prominent. The lower panels show a magnification of the indicated area (squares) in the upper panel (B, C, and L). Red arrows show exacerbated foot process effacement in Pod KO mice (E and I). *n*=6–9 (D); 5–8 (F); 4–7 (G); 4–8 (H); 4–7 (J); 5–7 (K); 5–7 (M) in each group. Bars: 1 mm (upper in B); 100 *μ*m (lower in B); 20 *μ*m (upper in C); 5 *μ*m (lower in C); 1 *μ*m (E and I); 100 *μ*m (upper in L); 50 *μ*m (lower in L). ACR, albumin-to-creatinine ratio; CTRL, control; DAPI, 4′,6-diamidino-2-phenylindole; DOCA, deoxicorticosterone acetate; GIT2, GIT ArfGAP2; KO, knockout; SBP, systolic BP; UN, urea nitrogen.

### Podocyte-Specific *Git2*-Deficient Mice Exhibited Increased Susceptibility to Proteinuria

To study the role of GIT2 in glomerular function, we initially generated systemic *Git2*-deficient (knockout [KO]) mice (Supplemental Figure 1, A–C, and Supplemental Methods). *Git2* KO mice did not develop proteinuria up to 6 months of age (Supplemental Figure 1D), and their glomeruli showed normal morphology (Supplemental Figure 1E). When challenged with LPS, however, *Git2* KO mice showed significantly increased proteinuria (Supplemental Figure 1F) and foot process effacement (Supplemental Figure 1, G and H) as compared with control littermates. By contrast, glomerular basement membrane thickness and fenestra of the glomerular endothelial cells were comparable between groups. To determine whether the above phenotype is attributable to GIT2 deficiency in podocytes, we next established podocyte-specific *Git2* KO mice by crossing *Git2* floxed mice with *Nphs2 Cre* mice (Supplemental Figure 2A and Supplemental Methods). KO mice were confirmed by PCR-based genotyping (Supplemental Figure 2B). Immunofluorescence staining using primary outgrowth of isolated glomeruli showed podocyte-specific GIT2 deletion in KO mice (Supplemental Figure 2C). By immunoblotting, glomerular GIT2 expression was significantly reduced by 47%±9% in KO mice compared with age-matched controls, consistent with the effective knockout in podocytes (Supplemental Figure 2, D and E).

First, we induced proteinuria by LPS and monitored urinary albumin-to-creatinine ratio (UACR) up to 48 hours. In control mice, UACR levels peaked at 24 hours and declined subsequently. By contrast, KO mice showed sustained high levels of proteinuria, resulting in a significantly higher UACR at 48 hours compared with controls (Figure 1D) (NSC23766-treated KO mice are discussed below). Consistently, quantification of foot process width showed significantly higher podocyte foot process effacement at 48 hours compared with controls (Figure 1, E and F), analogous to systemic KO mice.

To study the effect of GIT2 deficiency in chronic podocyte injury, we next used a salt-sensitive hypertension model where mice were uninephrectomized and treated with deoxicorticosterone acetate (DOCA) and salt for 14 days. In both KO and control mice, systolic BP and the ratio of heart weight to body weight were increased similarly at day 14 by uninephrectomy–DOCA/salt (Figure 1G and Supplemental Figure 3A). Consistent with a previous report,^29^ uninephrectomy–DOCA/salt–treated mice developed hypokalemia, but there was no significant difference in serum electrolytes between KO mice and controls (Supplemental Figure 3, B–D). Uninephrectomy–DOCA/salt induced a significant increase of UACR at day 14 in both mouse groups. KO mice tended to have more severe proteinuria than controls, although the difference did not reach statistical significance because of a large variability (Figure 1H). Foot process effacement was significantly exacerbated in KO mice, compared with controls (Figure 1, I and J). Serum urea nitrogen was significantly higher in uninephrectomy–DOCA/salt–treated KO mice compared with controls (Figure 1K). Consistent with a worse kidney function, KO mice showed more protein casts with tubular atrophy and leukocyte infiltration assessed by periodic acid–Schiff staining (Figure 1, L and M). Taken together, the results indicate that GIT2 protects podocytes and glomerular permselectivity against injury *in vivo*.

### *GIT2* KD Activated Rac1 and Caused Morphological Changes in Podocytes

**Figure 2:**
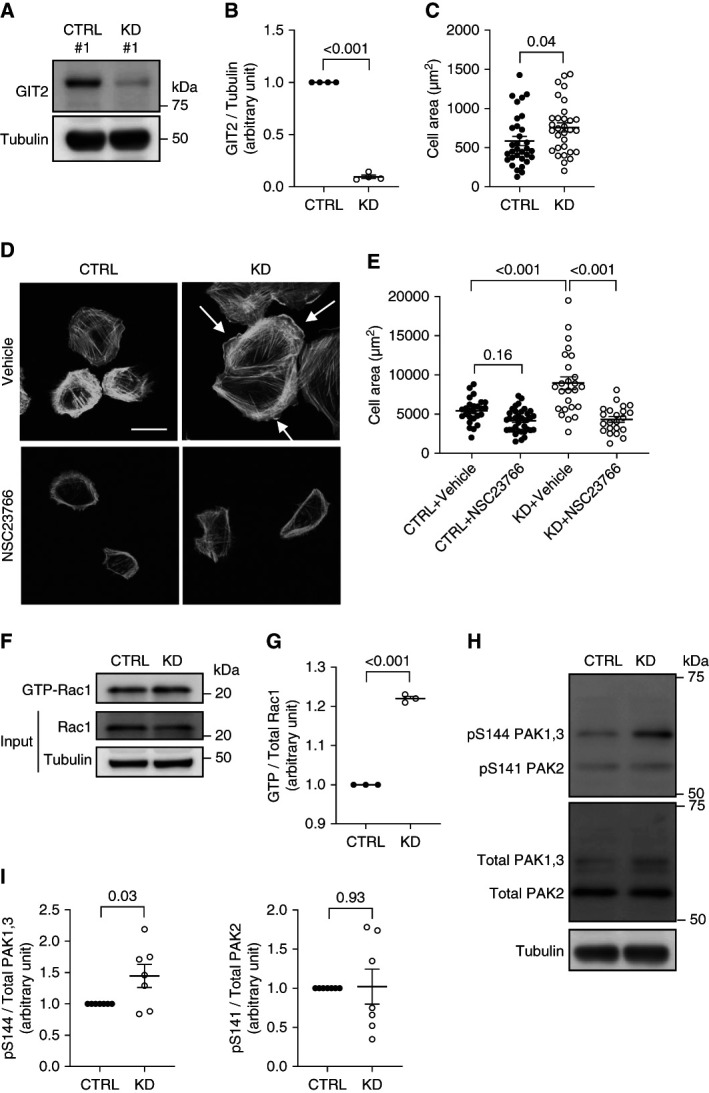
**GIT2 KD activates Rac1 and causes morphological changes in podocytes.** (A) Representative immunoblots for GIT2 and tubulin of cultured human podocytes with GIT2 KD (KD 1) and their controls (CTRL 1). (B) Densitometric quantification of GIT2 protein levels normalized to tubulin in (A). (C) Quantification of cell-spreading assay. Cell area after 2 hours of plating on laminin-521–coated coverslips is shown. (D) Representative images of phalloidin staining of differentiated GIT2 KD and control podocytes. Cells were treated with vehicle (upper) or 25 *µ*M NSC23766 (lower) for 4 days. Lamellipodia were extensive in vehicle-treated GIT2 KD podocytes (arrows) and inhibited by NSC23766. (E) Quantification of the cell area in (D). (F) Lysates from GIT2 KD and control podocytes were subjected to pull-down with GST-CRIB beads to detect active Rac1. Representative immunoblots for GTP-bound (active) forms of total Rac1 with tubulin are shown. (G) Densitometric quantification of active Rac1 normalized to its total expression in (F). (H) Representative immunoblots for pY and total PAK protein with tubulin. (I) Densitometric quantification of pY PAK protein normalized to total expression in (H). *n*=4 (B); 31 or 32 (C); 22–36 (E); 3 (G); 7 (I) in each group. Bar: 50 *μ*m (D). KD, knockdown; PAK, P21-activated kinase; pY, phosphorylated; Rac1, Ras-related C3 botulinum toxin substrate 1.

To understand the mechanisms by which GIT2 protects podocytes against injury, we knocked down *GIT2* in immortalized podocytes by shRNA. Densitometric analysis of immunoblots confirmed that GIT2 protein expression was significantly decreased by 90%±2% in KD podocytes compared with controls (Figure 2, A and B). When cells were plated on a laminin-521–coated surface for 2 hours, the cell surface area was significantly larger for GIT2 KD podocytes than controls, consistent with a spreading phenotype of KD cells (Figure 2C). When cells were further differentiated, GIT2 KD podocytes showed distinct lamellipodia at the cell edge, accompanied by a significantly larger cell area (Figure 2, D and E). To exclude the possibility that the phenotype was from off-targeting effects, we tested another shRNA probe, which targeted a different region of *GIT2* (Supplemental Figure 4A), and obtained analogous results to the first shRNA (Supplemental Figure 4B).

Lamellipodium formation and cell spreading are the well-known cellular phenotypes associated with Rac1 activation.^30^ Thus, we next studied whether gene silencing of *GIT2* affects the Rac1 activity. GST-CRIB pull-down assay showed a significant increase of Rac1-GTP (active Rac1) by 1.2-fold in GIT2 KD podocytes compared with controls (Figure 2, F and G). Similarly, phosphorylation of P21-activated kinase 1/3, which is known to be downstream of Rac1 activation,^29,31^ was significantly upregulated in GIT2 KD podocytes as assessed by immunoblotting (Figure 2, H and I). Finally, the morphological alterations in GIT2 KD podocytes were completely abolished by the Rac1 inhibitor, NSC23766 (Figure 2, D and E). As expected, NSC23766 also inhibited lamellipodia in control cells. Taken together, the results indicate that GIT2 suppresses Rac1 activity in podocytes and the gene silencing of *GIT2* results in the activation of Rac1 and its downstream signaling, leading to lamellipodium formation and cell spreading.

To determine whether a worsened phenotype of LPS-treated and uninephrectomy–DOCA/salt–treated KO mice was due to Rac1 activation, we treated them with subcutaneous injection of NSC23766. In both proteinuric disease models, the pharmacological inhibition of Rac1 significantly improved UACR (Figure 1, D and H) and foot process effacement in KO mice (Figure 1, E, F, I, and J). NSC23766 attenuated tubulointerstitial damage (Figure 1, L and M) in uninephrectomy–DOCA/salt–treated KO mice although serum urea nitrogen was not improved (Figure 1K). The results support that the protective effect of GIT2 in podocytes is likely dependent on Rac1.

### GIT2 Was Recruited to Focal Adhesions in a Tension-Dependent Manner in Podocytes

**Figure 3:**
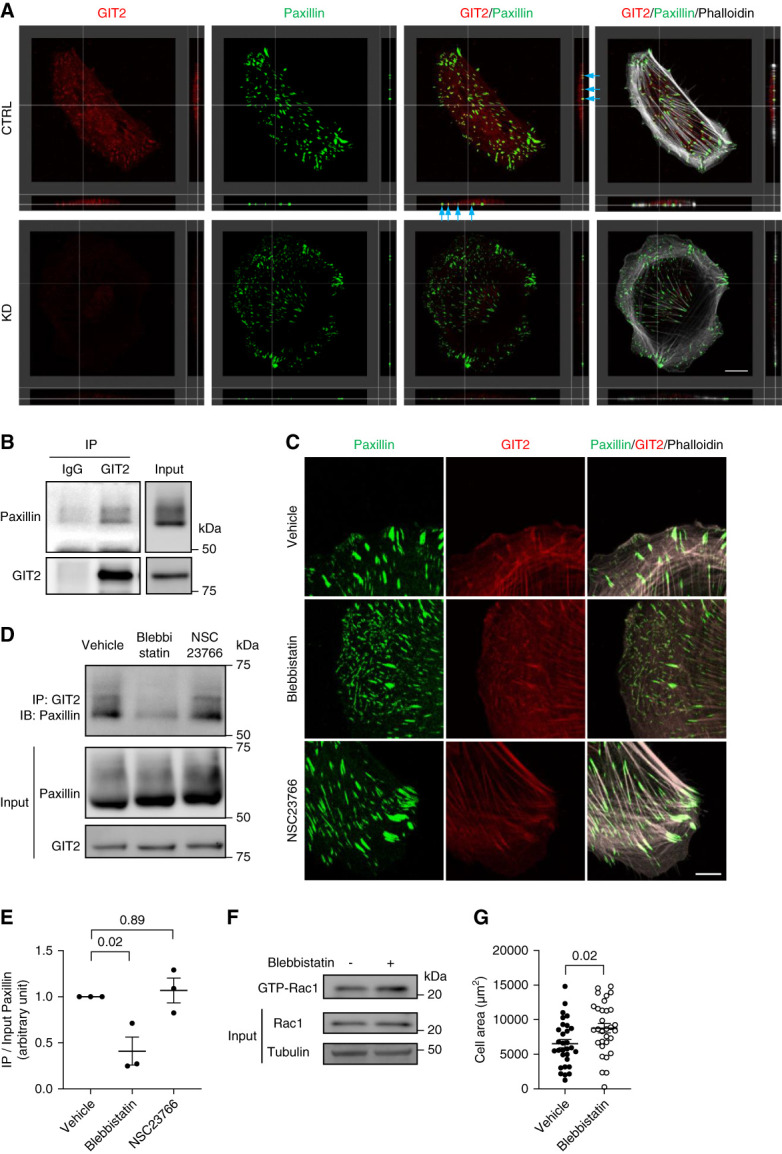
**Tension-dependent recruitment of GIT2 to focal adhesions in podocytes.** (A) Representative images of the immunofluorescence staining for GIT2 (red), paxillin (green), and phalloidin (gray) of differentiated cultured podocytes with GIT2 KD and controls. Reconstructed cross (right) and longitudinal (below) sections are shown. GIT2 colocalized with paxillin at the basolateral membrane in control cells (arrows) while GIT2 staining overlapping with paxillin was absent in KD cells. (B) Cell lysates were immunoprecipitated with anti-GIT2 antibody followed by immunoblotting for paxillin to determine the interaction between proteins. Rabbit IgG was used as a negative control. Representative immunoblots are shown. (C) Representative images of the immunofluorescence staining for paxillin (green), GIT2 (red), and phalloidin (gray) of cultured podocytes treated with either 50 *µ*M blebbistatin, NSC23766, or vehicle for 5 minutes. (D) Representative images of immunoprecipitation with anti-GIT2 antibody followed by immunoblotting for paxillin using lysates from cells treated with 12.5 *µ*M blebbistatin, NSC23766, or vehicle for 16 hours. (E) Quantification of paxillin immunoprecipitated by anti-GIT2 antibody normalized to total paxillin in (D). (F) Representative immunoblots for active and total Rac1 with tubulin. Podocytes were treated with 12.5 *µ*M blebbistatin for 30 minutes, and lysates were subjected to pull-down with GST-CRIB beads. (G) Quantification of the cell area after treatment with either 50 *µ*M blebbistatin or vehicles for 1 hour. *n*=3 (E); 30 or 31 (G) in each group. Bars: 20 *μ*m (A); 10 *μ*m (C).

To better understand how GIT2 functions in podocytes, we next studied the subcellular localization of endogenous GIT2. Immunofluorescence staining showed a clear colocalization of GIT2 and the focal adhesion protein paxillin at the basolateral membrane (Figure 3A), suggesting that GIT2 localizes in the focal adhesion complex. GIT2 staining overlapping with paxillin was absent in GIT2 KD cells (Figure 3A), confirming the specificity of this staining. The finding is consistent with the structure of GIT2, which contains the focal adhesion–targeting domain at the C terminus.^32,33^ Paxillin coimmunoprecipitated with GIT2, further confirming that these two proteins are in the same complex (Figure 3B).

Formation of focal adhesions is known to be triggered by cellular tension.^34^ To determine whether GIT2 is recruited to focal adhesions by cytoskeletal tension, we treated podocytes with the myosin II ATPase inhibitor blebbistatin. Blebbistatin markedly decreased focal adhesion size and disrupted GIT2 colocalization with paxillin (Figure 3C). Similarly, paxillin coimmunoprecipitation with GIT2 was markedly reduced by blebbistatin (Figure 3, D and E). By contrast, pharmacological inhibition of Rac1 by NSC23766 did not affect GIT2 colocalization/coimmunoprecipitation with paxillin (Figure 3, C–E), indicating that GIT2 acts upstream of Rac1. Interestingly, blebbistatin treatment markedly increased Rac1 activity (Figure 3F) and cell area (Figure 3G), mimicking GIT2 KD. The findings support that tension-dependent recruitment of GIT2 to the focal adhesions is required for its function to suppress local Rac1 activity.

### Downregulation of *GIT2* Promoted Focal Adhesion Turnover and Cell Motility in Podocytes

**Figure 4:**
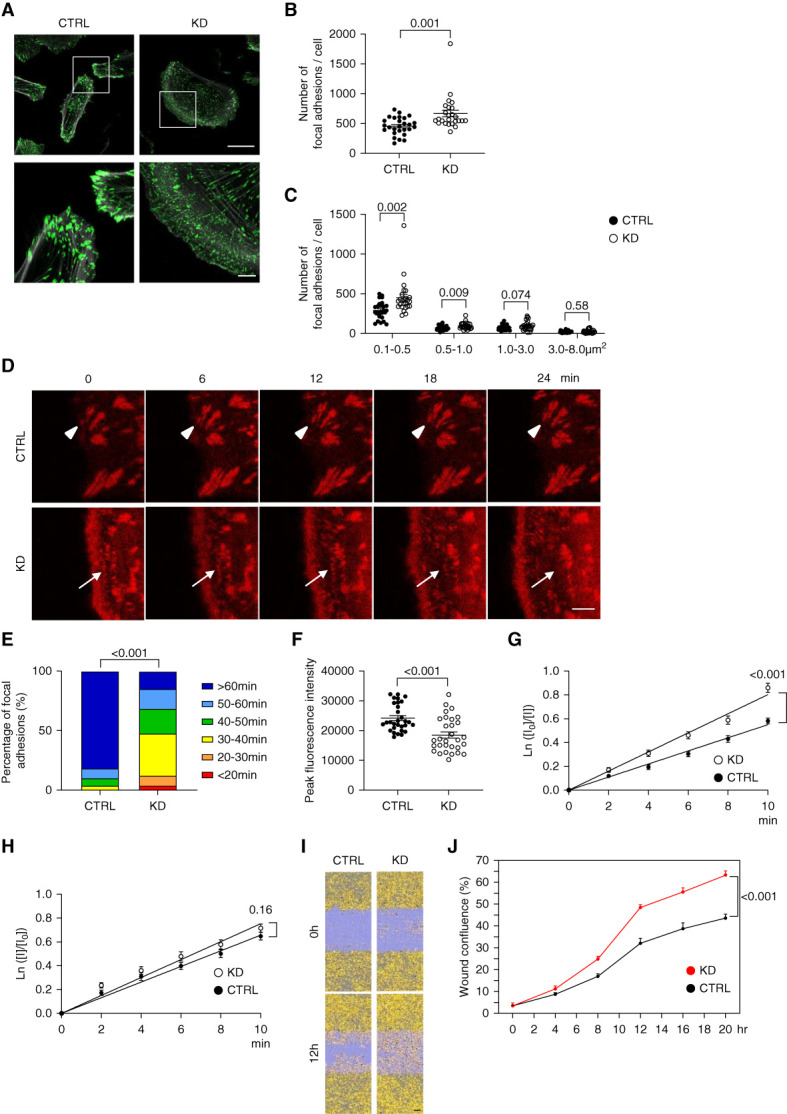
**Accelerated focal adhesion turnover and cell motility in GIT2 KD podocytes.** (A) Representative images of the immunofluorescence staining for paxillin (green) and phalloidin (gray) of differentiated cultured podocytes with GIT2 KD and controls. The lower panels show a magnification of the indicated area (white squares) in the upper panels. (B) Quantification of the total number of focal adhesions per cell in (A). (C) Quantification of the number of focal adhesions, which were categorized into four groups by size (0.1–0.5, 0.5–1.0, 1.0–3.0, and 3.0–8.0 *µ*m^2^), per cell in (A). (D) Representative images of the time-lapse confocal microscopy. Podocytes were transiently transfected mRFP-tagged paxillin. Most focal adhesions labeled by mRFP-paxillin in GIT2 KD podocytes are short-lived (arrows) while matured in controls (arrowheads). (E) Distribution of focal adhesion lifetime in (D) is shown. (F) Quantification of the peak focal adhesion intensity in (D). (G and H) Quantification of focal adhesion disassembly (G) and assembly (H) rates in (D) are shown. (I) Representative images of the time-lapse phase contrast microscopy showing a scratch wound healing assay. Initial wound region (blue) and the area occupied by cells (yellow) are shown. (J) Quantification of podocyte migration by wound confluence in (I). *n*=25 or 27 cells in two independent experiments (B and C); 48 focal adhesions in three cells (E); 30 focal adhesions in three cells (F); 20–30 focal adhesions in three cells (G and H); 5 (J) in each group. Bars: 50 *μ*m (upper in A); 10 *μ*m (lower in A); 5 *μ*m (D); 100 *μ*m (I).

On the basis of the localization of GIT2 in focal adhesions and the spreading phenotype of GIT2 KD podocytes, we hypothesized that GIT2 may control focal adhesion dynamics in podocytes. To test this hypothesis, we quantified the number and size of focal adhesions (as defined by paxillin-positive areas). The total number of focal adhesions per cell was more abundant in GIT2 KD podocytes than in controls (Figure 4, A and B). When the focal adhesions were divided into four groups by size, nascent focal adhesions (defined as <1.0 *µ*m^2^)^31,35^ were markedly increased in GIT2 KD podocytes compared with controls (Figure 4C).

Focal adhesions are dynamic complex structures. The coordinated formation (assembly), maturation, and degradation (disassembly) is collectively called turnover and is required for the transduction of integrin-mediated signals between extracellular matrix and intracellular actin cytoskeleton.^31,34^ To explore the underlying mechanism of the increase in nascent focal adhesions in GIT2 KD, we monitored focal adhesion turnover using time-lapse confocal microscopy in cells transfected with mRFP-tagged paxillin. Consistent with the staining of fixed cells, GIT2 KD podocytes had a large number of mRFP-labeled focal adhesion clusters at the cell periphery, accompanied by vigorous membrane ruffle formation (Figure 4D and Supplemental Movie 1). By contrast, most of the mRFP-labeled puncta at the edge of control podocytes grew in size (matured) and remained stationary (Figure 4D and Supplemental Movie 1). Quantification confirmed that, compared with controls, focal adhesion lifetime in GIT2 KD podocytes was significantly shorter (Figure 4E), focal adhesion peak fluorescence intensity (just before degradation) was lower (Figure 4F), and the focal adhesion disassembly rate was higher (Figure 4G), whereas the assembly rate did not differ between groups (Figure 4H). These results indicate that focal adhesion turnover is accelerated by GIT2 KD.

We next examined whether the increased focal adhesion turnover in GIT2 KD podocytes was dependent on Rac1. GIT2 KD podocytes treated with the Rac1 inhibitor NSC23766 showed more abundant mature focal adhesions (Supplemental Figure 5A and Supplemental Movie 2), which resulted in a significantly longer focal adhesion lifetime than the vehicle-treated group (Supplemental Figure 5B). The low peak intensity and accelerated disassembly rate of focal adhesions in GIT2 KD podocytes were both reversed by NSC23766 (Supplemental Figure 5, C and D), while there was no obvious change in the assembly rate (Supplemental Figure 5E). Thus, we concluded that the accelerated focal adhesion turnover induced by GIT2 KD was mediated by Rac1 activation in podocytes.

Consistent with the accelerated focal adhesion turnover, cell motility as determined by wound healing assay was significantly increased in GIT2 KD podocytes compared with controls (Figure 4, I and J, and Supplemental Movie 3). These findings indicate that GIT2 plays a critical role in maintaining focal adhesion stability by suppressing the Rac1 activity and that the loss of GIT2 results in accelerated focal adhesion turnover and increased motility of podocytes.

### Downregulation of GIT2 Activated p130Cas by Disrupting PTP1B Function at Focal Adhesions

**Figure 5:**
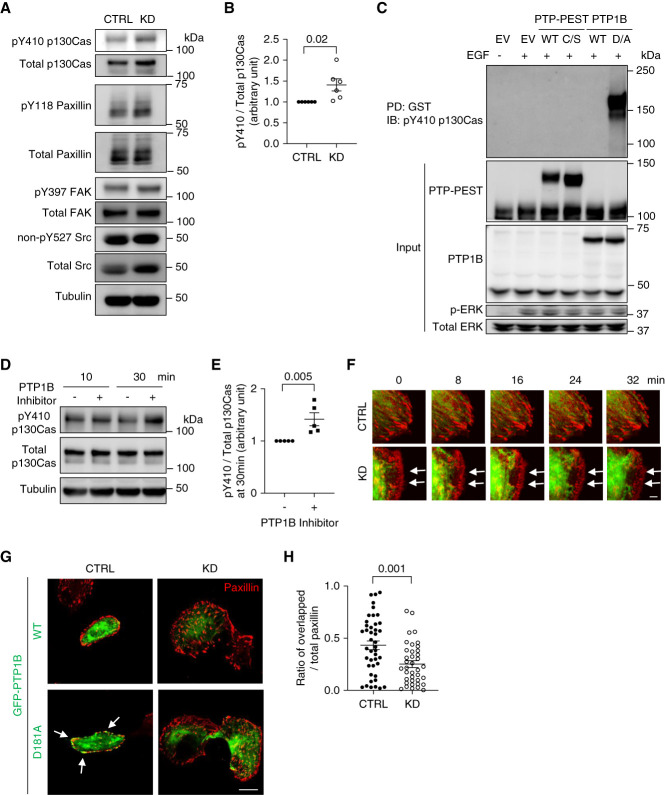
**Downregulation of GIT2 activates p130Cas by disrupting PTP1B function at focal adhesions.** (A) Representative immunoblots for pY and total abundance of focal adhesion component proteins with tubulin of cultured podocytes with GIT2 KD and control. (B) Densitometric quantification of pY410 normalized to total expression of p130Cas in (A). (C) Representative immunoblots for substrate-trapping assay. HEK293 cells were transiently transfected plasmids containing an EGF receptor with pEBG-tagged PTP-PEST WT, PTP-PEST-C231S (C/S), PTP1B WT, PTP1B-D181A (D/A), or EV. After 100 ng/ml EGF stimulation overnight, cell lysates were subjected to pull-down with GST followed by immunoblotting for pY410 p130Cas. (D) Representative immunoblots for pY and total p130Cas with tubulin of cultured podocytes treated with 50 *µ*M PTP1B inhibitor for indicated time. (E) Densitometric quantification of pY410 normalized to total p130Cas expression after 30 minutes of PTP1B treatment in (D). (F) Representative images of the time-lapse confocal microscopy. Podocytes were transiently transfected mRFP-tagged paxillin and GFP-tagged PTP1B WT. The localization of GFP-PTP1B WT to the ruffles seems absent (delayed) in GIT2 KD podocytes (arrows). (G) Representative images of the immunofluorescence staining for endogenous paxillin (red) and GFP-PTP1B (green). Podocytes were transiently transfected either GFP-tagged PTP1B WT or substrate-trapping mutant, PTP1B-D181A. PTP1B-D181A stabilizes the enzyme–substrate complex and thus is suitable for the analysis of PTP1B localization. Both PTP1B WT and PTP1B-D181A preferentially localized to ER. A part of PTP1B-D181A was distributed at paxillin-positive focal adhesions in control cells (arrows) while absent in GIT2 KD cells (lower panels). By contrast, PTP1B WT was rarely distributed at focal adhesions in both cells due to prompt dissociation after enzyme–substrate reaction (upper panels). (H) Quantification of the paxillin area overlapped with GFP-PTP1B-D181A normalized to total paxillin area in (G). Analyses were focused on the cell edge. *n*=6 (B); 5 (E); 38 or 43 (H) in each group. Bars: 5 *μ*m (F); 20 *μ*m (G). ER, endoplasmic reticulum; EV, empty vector; FAK, focal adhesion kinase; PTP, protein tyrosine phosphatases; WT, wild-type.

In the next set of experiments, we studied the molecular mechanisms of accelerated focal adhesion turnover in GIT2 KD. High focal adhesion turnover is generally associated with activation of various focal adhesion proteins.^31^ We first screened the activation status of the major focal adhesion proteins in GIT2 KD podocytes using phospho-specific antibodies that detect activated proteins. Among them, activation of p130Cas (detected by pY410-p130Cas) was most prominent, whereas the activities of other focal adhesion proteins studied (paxillin, focal adhesion kinase [FAK], and Src) were marginally increased or unchanged (Figure 5, A and B). It has been shown that p130Cas activates Rac1 using DOCK180 and Crk.^36–38^ Thus, we reasoned that p130Cas activity may be modulated by GIT2.

Tyrosine phosphorylation of proteins can be modulated by tyrosine kinases or phosphatases. Given that the two major kinases for p130Cas (Src and FAK)^38–40^ were not activated in GIT2 KD podocytes (Figure 5A), we postulated that protein tyrosine phosphatases (PTP) may be implicated. PTP-PEST and PTP1B are prototypical tyrosine phosphatases in podocytes.^41^ Using the substrate-trapping mutants, we found that PTP1B, but not PTP-PEST, binds to pY410-p130Cas (Figure 5C), suggesting that pY410 of p130Cas can be a substrate of PTP1B. Indeed, when control podocytes were incubated with the PTP1B inhibitor, pY410-p130Cas increased significantly (Figure 5, D and E).

It has been shown that while the PTP1B is predominantly expressed in the endoplasmic reticulum (ER), it relocalizes to focal adhesions by a microtubule-mediated mechanism, thereby controlling focal adhesion dynamics.^42^ When control podocytes were transfected with PTP1B (GFP-tagged) and mRFP-paxillin and observed by time-lapse fluorescence microscopy, PTP1B reached focal adhesions (detected as mRFP-paxillin clusters) at the membrane ruffle (Figure 5F and Supplemental Movie 4). By contrast, in GIT2 KD podocytes, PTP1B remained in the ER area and did not reach the membrane ruffle/focal adhesions (Figure 5F and Supplemental Movie 4). When colocalization of PTP1B and paxillin was quantified in fixed cells using the substrate-trapping mutant, we found a marked reduction in GIT2 KD podocytes compared with controls (Figure 5, G and H). Taken together, we propose that GIT2 facilitates focal adhesion localization of PTP1B, thereby suppressing p130Cas activity and downstream Rac1 activation. Conversely, the absence of GIT2 impairs focal adhesion localization of PTP1B, leading to activation of p130Cas and Rac1 in focal adhesions.

### GIT2 Reconstitution Rescued the Phenotype of Podocytes with GIT2 KD

**Figure 6:**
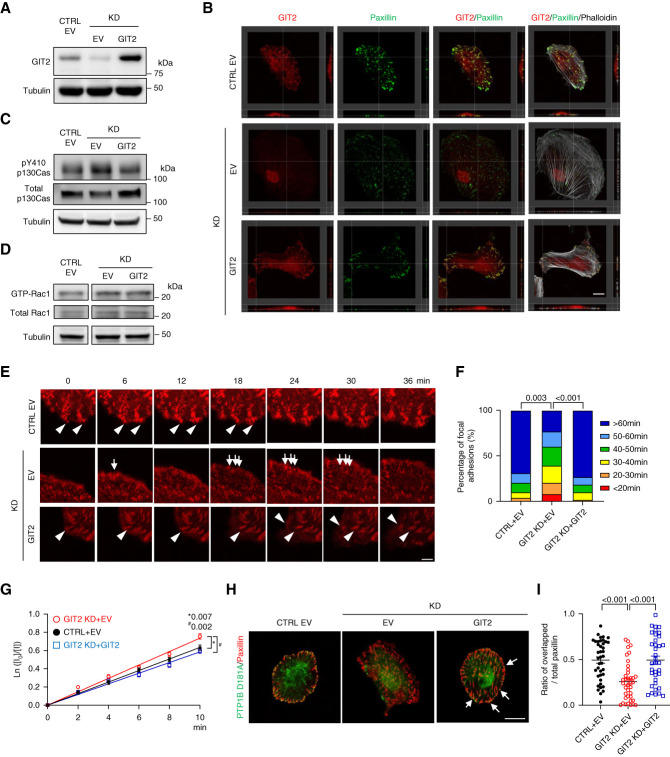

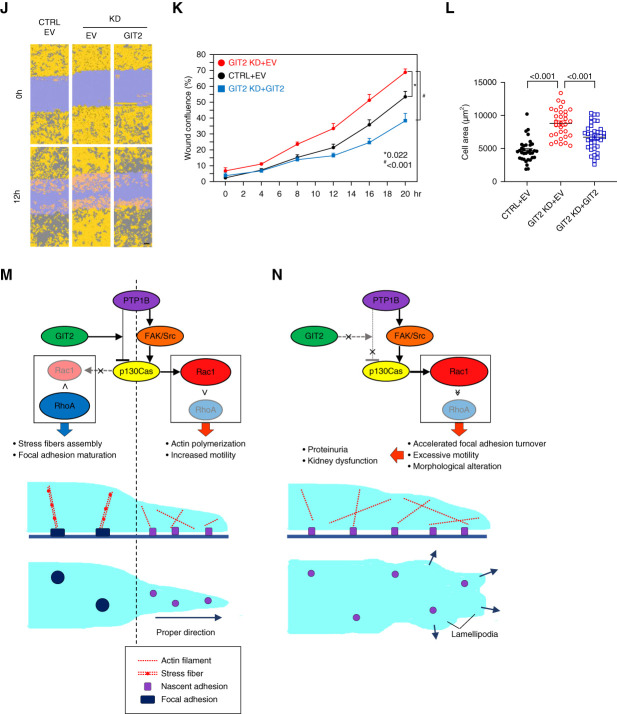
**GIT2 reconstitution rescues the phenotype of podocytes with GIT2 KD.** (A) Generation of GIT2-overexpressing podocytes. Either human GIT2 or EV was transfected into podocytes using lentiviral approach. Representative immunoblots for GIT2 and tubulin are shown. (B) Representative images of the immunofluorescence staining for GIT2 (red), paxillin (green), and phalloidin (gray) of GIT2 KD podocytes transfected either EV or GIT2 and EV-transfected control cells. Reconstructed cross (right) and longitudinal (below) sections are shown. Colocalization of GIT2 with paxillin at the basolateral membrane was restored in GIT2-overexpressing cells. (C) Representative immunoblots of pY410 and total p130Cas with tubulin. (D) Representative GST-CRIB pull-down followed by immunoblots of GTP-bound (active) forms of total Rac1 with tubulin. (E) Representative images of the time-lapse confocal microscopy. Podocytes were transiently transfected mRFP-tagged paxillin. Most focal adhesions labeled by mRFP-paxillin in GIT2 KD podocytes were short-lived (arrows) while matured in control and GIT2-overexpressing cells (arrowheads). (F) Distribution of focal adhesion lifetime in (E). (G) Quantification of focal adhesion disassembly in (E). (H) Podocytes were transiently transfected substrate-trapping mutant, GFP-PTP1B-D181A. Representative images of the immunofluorescence staining for endogenous paxillin (red) and GFP-PTP1B-D181A (green) are shown. Distribution of PTP1B-D181A at paxillin-positive focal adhesions was restored in GIT2-overexpressing cells (arrows). (I) Quantification of the paxillin area overlapped with GFP-PTP1B-D181A normalized to total paxillin area in (H). Analyses were focused on the cell edge. (J) Representative images of the time-lapse phase contrast microscopy showing a scratch wound healing assay. Initial wound region (blue) and the area occupied by cells (yellow) are shown. (K) Quantification of podocyte migration by wound confluence in (J). (L) Quantification of the cell area of differentiated podocytes. (M and N) A model for Rac1 regulation by GIT2 in podocytes. (M) While PTP1B directly dephosphorylates/inactivates p130Cas, it also dephosphorylates Src pY527, and subsequent increase in FAK pY397 activates p130Cas. GIT2 facilitates translocation of the tyrosine phosphatase, PTP1B, to focal adhesions where it dephosphorylates p130Cas (Y410). This results in the suppression of the local Rac1 activity, and dominant RhoA signaling contributes to stress fiber formation and focal adhesion maturation. (N) GIT2 deficiency disrupts the direct pathway of PTP1B toward p130Cas, allowing the indirect (using Src/FAK) pathway to be dominant, thereby promoting p130Cas phosphorylation/activation. This leads to Rac1 activation, resulting in accelerated focal adhesion turnover and morphological alteration. *n*=48 focal adhesions in three cells (F); 20–30 focal adhesions in three cells (G); 36–40 (I); 7–11 (K); 31–37 (L) in each group. Bars: 20 *μ*m (B); 5 *μ*m (E); 20 *μ*m (H); 100 *μ*m (J).

To validate the causal relationship between GIT2 deletion and the observed phenotype in podocytes, we transfected a shRNA-resistant human GIT2 into GIT2 KD podocytes and established a stable cell line. Overexpression of GIT2 was confirmed by immunoblotting (Figure 6A). The protein abundance of its paralog, GIT ArfGAP1, was similar among the groups (Supplemental Figure 6), suggesting that the effect of GIT2 on GIT ArfGAP1 expression is minimal. Colocalization of GIT2 with paxillin at the basolateral membrane was restored in GIT2-overexpressing cells (Figure 6B). The following phenotypes observed in GIT2 KD podocytes were reversed by re-expression of GIT2: activation of p130Cas (Figure 6C), activation of Rac1 (Figure 6D), shorter lifetime of focal adhesions (Figure 6, E and F, and Supplemental Movie 5), increased disassembly rate of focal adhesions (Figure 6G), reduced colocalization of PTP1B and paxillin (Figure 6, H and I), increased migration (Figure 6, J and K), and increased cell size (Figure 6L).

## Discussion

We and others showed previously that hyperactivation of the Rho GTPase Rac1 is detrimental to podocyte health and causes proteinuria.^9,10^ In this study, we demonstrated that the focal adhesion protein GIT2 suppresses Rac1 activity in focal adhesions in podocytes, thereby protecting against injury and proteinuria. Furthermore, we showed that GIT2 facilitates translocation of the tyrosine phosphatase PTP1B to focal adhesions where it dephosphorylates p130Cas (Y410), and this results in the suppression of the local Rac1 activity (Figure 6M).

To the best of our knowledge, this is the first study to demonstrate the protective role of GIT2 in podocytes. Worsened proteinuria and foot process effacement of KO mice in two proteinuric disease models^43,44^ strengthen the importance of GIT2 after podocyte injury. Furthermore, the improvement by NSC23766 in KO mice indicates that the Rac1 pathway is responsible for the phenotype. Rac1 inhibitors may also have antihypertensive effects by inhibition of reactive oxygen species in the central nervous system.^12,45^ Indeed, the mean BP of NSC23766-treated mice was not significantly higher than before uninephrectomy–DOCA/salt treatment (Figure 1G). Nonetheless, when individual mice were examined, NSC23766 reduced proteinuria and foot process width in several mice whose BP was clearly elevated. Thus, the antiproteinuric effect of NSC23766 is likely mediated by Rac1 inhibition in podocytes.

Although several studies demonstrated a functional link between GIT2 and Rac1, how GIT2 affects the Rac1 activity seems cell type–specific.^46^ In human endothelial cells and mouse neutrophils, GIT2 seemed to activate Rac1.^18,47^ By contrast, GIT2 inhibited Rac1 in epithelial cancer cells, such as HeLa and MCF10A, although the mechanism of Rac1 inhibition was not studied in detail.^48,49^ Our results are consistent with the latter and demonstrate that GIT2 is inhibitory to Rac1 activation in glomerular epithelial cells, podocytes.

It has been shown that p130Cas activates Rac1 in focal adhesions upon tyrosine phosphorylation by tyrosine kinases, such as FAK and Src.^38–40^ However, data have been conflicting regarding which tyrosine phosphatase dephosphorylates (thereby deactivates) p130Cas.^36,50,51^ Using a substrate-trapping assay, we clearly showed that p130Cas is a substrate of PTP1B in podocytes. PTP1B is bound to the ER, and its catalytic domain faces the cytosol.^42^ Microtubule-dependent extension of the ER tubules toward the cell periphery allows PTP1B to dephosphorylate its substrates located at focal adhesions. We demonstrated that GIT2 is required for the PTP1B translocation to focal adhesions where it dephosphorylates p130Cas, thereby inhibiting Rac1 (Figure 6M). Further studies are required to determine whether GIT2 directly binds to and moves with PTP1B to focal adhesions or it functions as a scaffold to retain PTP1B at focal adhesions.

Of note, PTP1B is also known to activate Src by dephosphorylating pY527, which leads to activation of FAK and p130Cas.^26,52^ Thus, PTP1B may directly inactivate, but indirectly activate (using Src/FAK), p130Cas (Figure 6M). This could explain why PTP1B has been reported to activate or inactivate Rac1, depending on the study.^26,52,53^ We postulate that GIT2 deficiency disrupts the direct inhibition of p130Cas by PTP1B, allowing the indirect (using Src/FAK) pathway to dominate, thereby promoting phosphorylation/activation of p130Cas (Figure 6N).

Data regarding the subcellular localization of endogenous GIT2 are limited. Studies from two groups have shown that endogenous GIT2 localizes at focal adhesions in adherent cells,^48,49,54,55^ but the data were conflicting whether GIT2 recruitment to focal adhesions was Rac1 or RhoA dependent. The current results support that GIT2 acts downstream of RhoA and upstream of Rac1 (Figure 3, C–E). A tight regulation of RhoA and Rac1 is critical in podocyte function. RhoA regulates cytoskeletal tension using myosin II and forms stress fibers, allowing cells to be static, whereas Rac1 forms lamellipodia and allows cells to be motile.^30^ Because RhoA and Rac1 have opposing roles in actin cytoskeleton, it was previously believed that RhoA localizes at the cell rear and Rac1 at the cell front.^5,56^ However, more recent work has shown that they act in concert spatiotemporally. For example, RhoA regulates Rac1 using GTPase-activating proteins, including FilGAP and ARHGAP22.^57,58^ Our findings suggest that GIT2 modulates the signal transduction from RhoA to Rac1 at focal adhesions in podocytes.

In the Ju CKD Glom dataset from the Nephroseq, glomerular GIT2 mRNA was upregulated in many proteinuric diseases. However, the Nephroseq datasets do not allow the resolution at the single-cell level. Thus, we next studied single-nucleus RNA sequencing datasets and found that in two studies, the proportion of podocytes expressing GIT2 was decreased in human diabetic nephropathy.^27,28^ Nevertheless, in most available single-cell/nucleus RNA datasets, GIT2 in podocytes was not identified as a differentially expressed gene; and even when it was, the *P* value after adjustment was NS between proteinuric patients and healthy controls, possibly because of the small number of podocytes captured. Thus, available datasets indicate that, while glomerular GIT2 expression may increase in certain proteinuric diseases, it is likely decreased in podocytes at least in diabetic kidney diseases. Additional single-cell/nucleus RNA sequencing data and GIT2 antibodies which work for immunostaining in human kidney biopsies would be required for a definitive answer. It is tempting to speculate that GIT2 suppression might contribute to the development of nephropathy by inducing Rac1 activation in podocytes. Of note, we showed that GIT2 overexpression in KD podocytes restored Rac1 suppression, focal adhesion stability, and cell size to the levels of control cells. Thus, GIT2 could be viewed as a potential therapeutic target in diabetic nephropathy. Interestingly, in the mouse diabetic model, decreased *Git2* expression in podocytes was restored by sodium-glucose cotransporter 2 inhibition.^59^ There might be a previously unrecognized protective effect of sodium-glucose cotransporter 2 inhibitors through GIT2.

In summary, the current results provide a novel mechanism of Rac1 regulation by which GIT2 protects podocytes against injury. Stabilizing GIT2 or facilitating GIT2 localization to focal adhesions in podocytes may be an attractive therapeutic strategy in proteinuric kidney diseases.

## Supplementary Material

SUPPLEMENTARY MATERIAL

## Data Availability

All data are included in the manuscript and/or supporting information. Partial restrictions to the data and/or materials apply. Additional information is available from the corresponding author upon reasonable request.

## Supplemental Material

Supplemental Methods

Supplemental Figure 1. Systemic *Git2* deletion exacerbates proteinuria and podocyte foot process effacement after LPS-induced injury in mice.

Supplemental Figure 2. Generation of podocyte-specific *Git2*-deficient mice.

Supplemental Figure 3. Heart weight and serum electrolytes are comparable between podocyte-specific *Git2*-deficient and control mice in a salt-sensitive hypertension model.

Supplemental Figure 4. Gene silencing of GIT2 using another probe causes analogous phenotype, eliminating possible off-targeting effects.

Supplemental Figure 5. Pharmacological inhibition of Rac1 ameliorates accelerated focal adhesion turnover in GIT2 KD podocytes.

Supplemental Figure 6. Effect of GIT2 on its paralog GIT1 expression is minimal.

Supplemental Movie 1. Accelerated focal adhesion turnover in GIT2 KD podocytes.

Supplemental Movie 2. Pharmacological inhibition of Rac1 ameliorates accelerated focal adhesion turnover in GIT2 KD podocytes.

Supplemental Movie 3. Accelerated cell motility in GIT2 KD podocytes.

Supplemental Movie 4. Delayed localization of PTP1B to the ruffles in GIT2 KD podocytes.

Supplemental Movie 5. GIT2 reconstitution canceled accelerated focal adhesion turnover in podocytes with GIT2 KD.
