# Supplementary material for: Rac1 Suppression by the Focal Adhesion Protein GIT ArfGAP2 and Podocyte Protection
Source: J Am Soc Nephrol. 2025 Feb 28;36(6):1088–104. doi: 10.1681/ASN.0000000614 (PMC12147964; doi:10.1681/ASN.0000000614)
Supplement: SUPPLEMENTARY MATERIAL [file jasn-36-1088-s001.pptx]

## Slide 1
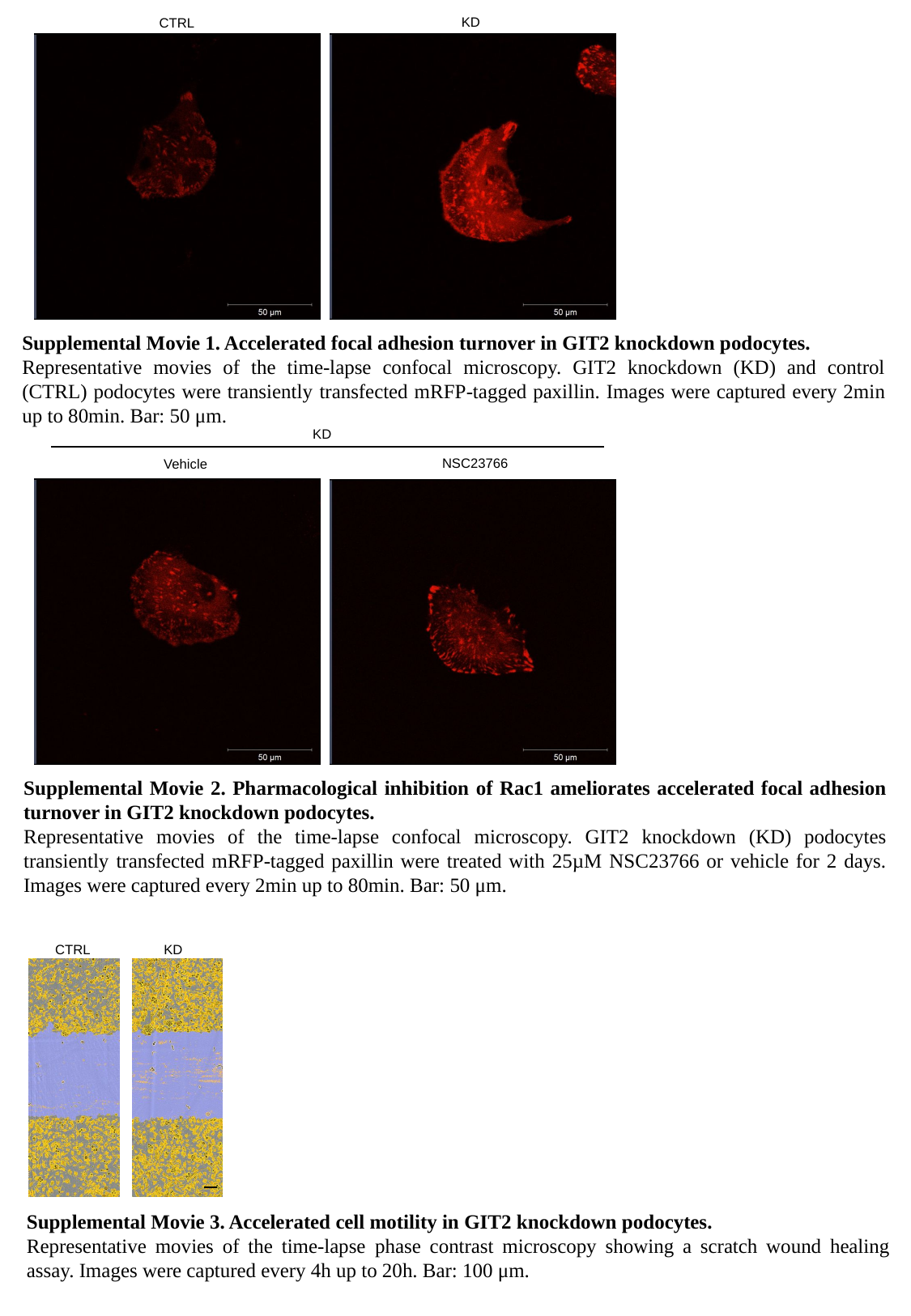

KD
CTRL
Supplemental Movie 1. Accelerated focal adhesion turnover in GIT2 knockdown podocytes.
Representative movies of the time-lapse confocal microscopy. GIT2 knockdown (KD) and control (CTRL) podocytes were transiently transfected mRFP-tagged paxillin. Images were captured every 2min up to 80min. Bar: 50 μm.
KD
NSC23766
Vehicle
Supplemental Movie 2. Pharmacological inhibition of Rac1 ameliorates accelerated focal adhesion turnover in GIT2 knockdown podocytes.
Representative movies of the time-lapse confocal microscopy. GIT2 knockdown (KD) podocytes transiently transfected mRFP-tagged paxillin were treated with 25µM NSC23766 or vehicle for 2 days. Images were captured every 2min up to 80min. Bar: 50 μm.
KD
CTRL
Supplemental Movie 3. Accelerated cell motility in GIT2 knockdown podocytes.
Representative movies of the time-lapse phase contrast microscopy showing a scratch wound healing assay. Images were captured every 4h up to 20h. Bar: 100 μm.

## Slide 2
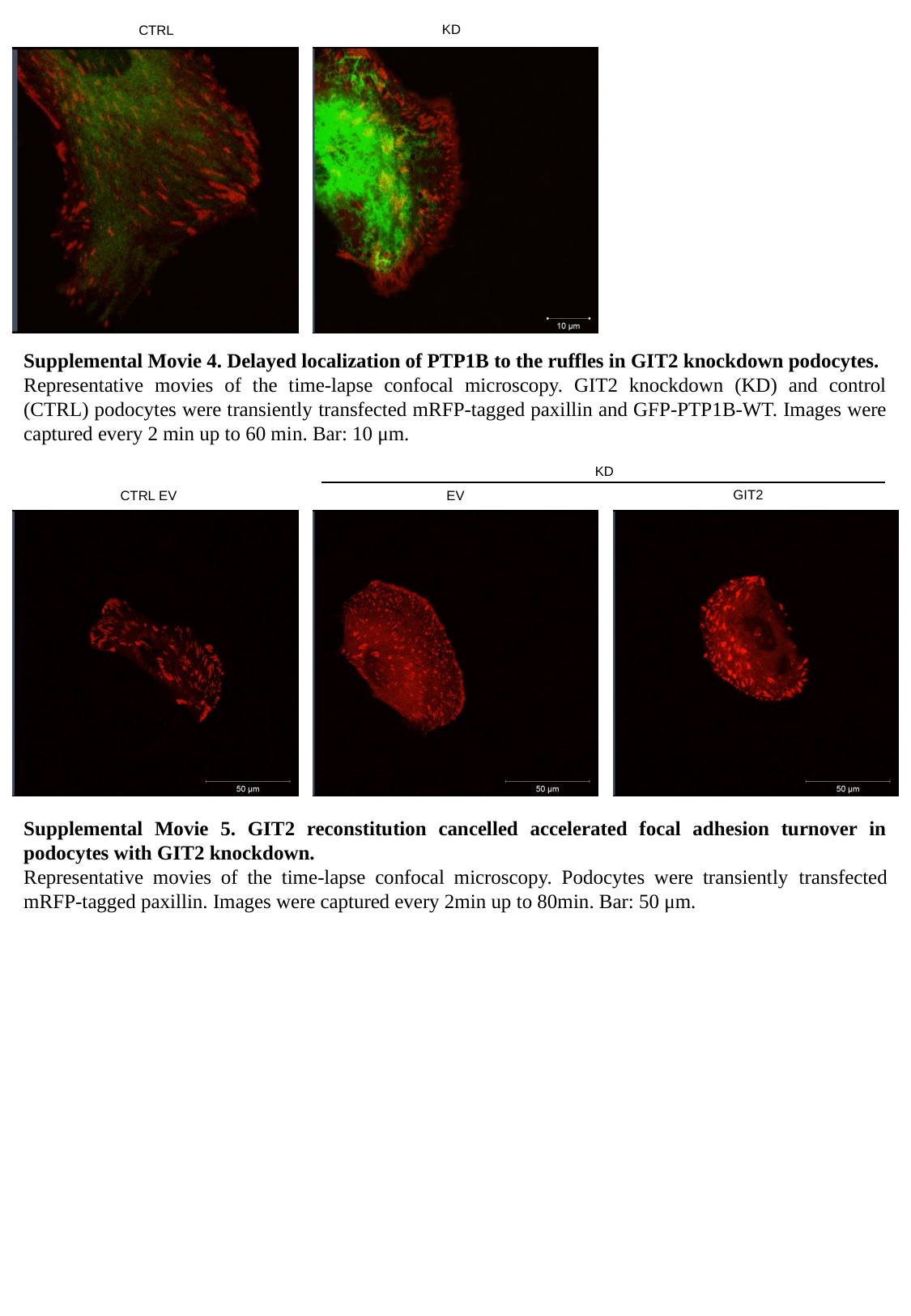

KD
CTRL
Supplemental Movie 4. Delayed localization of PTP1B to the ruffles in GIT2 knockdown podocytes.
Representative movies of the time-lapse confocal microscopy. GIT2 knockdown (KD) and control (CTRL) podocytes were transiently transfected mRFP-tagged paxillin and GFP-PTP1B-WT. Images were captured every 2 min up to 60 min. Bar: 10 μm.
KD
CTRL EV
EV
GIT2
Supplemental Movie 5. GIT2 reconstitution cancelled accelerated focal adhesion turnover in podocytes with GIT2 knockdown.
Representative movies of the time-lapse confocal microscopy. Podocytes were transiently transfected mRFP-tagged paxillin. Images were captured every 2min up to 80min. Bar: 50 μm.
