# Supplementary material for: Rac1 Suppression by the Focal Adhesion Protein GIT ArfGAP2 and Podocyte Protection
Source: J Am Soc Nephrol. 2025 Feb 28;36(6):1088–104. doi: 10.1681/ASN.0000000614 (PMC12147964; doi:10.1681/ASN.0000000614)
Supplement: SUPPLEMENTARY MATERIAL [file jasn-36-1088-s002.pdf]

ASN American Society of Nephrology | JASN Journal of the American Society of Nephrology | KIDNEY360 Accessing Our World From Every Angle | CJASN Clinical Journal of the American Society of Nephrology

ASN Journal Disclosure Form

As per ASN journal policy, I have disclosed any financial relationships or commitments I have held in the past 36 months as included below. I have listed my Current Employer below to indicate there is a relationship requiring disclosure. If no relationship exists, my Current Employer is not listed.

L. Aoudjit has nothing to disclose.

I understand that the information above will be published within the journal article, if accepted, and that failure to comply and/or to accurately and completely report the potential financial conflicts of interest could lead to the following: 1) Prior to publication, article rejection, or 2) Post-publication, sanctions ranging from, but not limited to, issuing a correction, reporting the inaccurate information to the authors' institution, banning authors from submitting work to ASN journals for varying lengths of time, and/or retraction of the published work.

Name: Lamine Aoudjit
Manuscript ID: JASN-2024-000997R1
Manuscript Title: Rac1 suppression by GIT2 and podocyte protection
Date of Completion: December 11, 2024
Disclosure Updated Date: May 21, 2024

ASN American Society of Nephrology JASN Journal of the American Society of Nephrology KIDNEY360 Accessing Our World From Every Angle CJASN Clinical Journal of the American Society of Nephrology

ASN Journal Disclosure Form

As per ASN journal policy, I have disclosed any financial relationships or commitments I have held in the past 36 months as included below. I have listed my Current Employer below to indicate there is a relationship requiring disclosure. If no relationship exists, my Current Employer is not listed.

K. Asano-Matsuda reports the following:
Employer: Department of Nephrology, Osaka University Graduate School of Medicine

I understand that the information above will be published within the journal article, if accepted, and that failure to comply and/or to accurately and completely report the potential financial conflicts of interest could lead to the following: 1) Prior to publication, article rejection, or 2) Post-publication, sanctions ranging from, but not limited to, issuing a correction, reporting the inaccurate information to the authors' institution, banning authors from submitting work to ASN journals for varying lengths of time, and/or retraction of the published work.

Name: Kana Asano-Matsuda
Manuscript ID: JASN-2024-000997R1
Manuscript Title: Rac1 suppression by GIT2 and podocyte protection
Date of Completion: December 8, 2024
Disclosure Updated Date: December 8, 2024

ASN American Society of Nephrology | JASN Journal of the American Society of Nephrology | KIDNEY360 Accessing Our World From Every Angle | CJASN Clinical Journal of the American Society of Nephrology

ASN Journal Disclosure Form

As per ASN journal policy, I have disclosed any financial relationships or commitments I have held in the past 36 months as included below. I have listed my Current Employer below to indicate there is a relationship requiring disclosure. If no relationship exists, my Current Employer is not listed.

Y. Isaka reports the following:
Employer: Osaka University Graduate School of Medicine; Consultancy: Kirin Co. Ltd.; Sanwa Kagaku Kenkyusyo Co.Ltd.; Research Funding: Kirin Co. Ltd., Zenyaku Kogyo Co., Ltd.; Advisory or Leadership Role: Kirin Co. Ltd.; Sanwa Kagaku Kenkyusyo Co.Ltd.; and Speakers Bureau: AstraZeneca plc; Kirin Co. Ltd.; Otsuka Pharmaceutical Co.Ltd.; Mitsubishi Tanabe Pharma; Kissei Pharmaceutical Co.Ltd.; Astellas Pharma Inc.; Sanwa Kagaku Kenkyusyo Co.Ltd.; Astellas Pharma Inc.

I understand that the information above will be published within the journal article, if accepted, and that failure to comply and/or to accurately and completely report the potential financial conflicts of interest could lead to the following: 1) Prior to publication, article rejection, or 2) Post-publication, sanctions ranging from, but not limited to, issuing a correction, reporting the inaccurate information to the authors' institution, banning authors from submitting work to ASN journals for varying lengths of time, and/or retraction of the published work.

Name: Yoshitaka Isaka
Manuscript ID: JASN-2024-000997R1
Manuscript Title: Rac1 suppression by GIT2 and podocyte protection
Date of Completion: December 8, 2024
Disclosure Updated Date: July 29, 2024

ASN American Society of Nephrology | JASN Journal of the American Society of Nephrology | KIDNEY360 Accessing Our World From Every Angle | CJASN Clinical Journal of the American Society of Nephrology

ASN Journal Disclosure Form

As per ASN journal policy, I have disclosed any financial relationships or commitments I have held in the past 36 months as included below. I have listed my Current Employer below to indicate there is a relationship requiring disclosure. If no relationship exists, my Current Employer is not listed.

S. Lemay reports the following:
Employer: Research Institute of the McGill University Health Centre; and Advisory or Leadership Role: Québec Society of Nephrology (Treasurer, unpaid).

I understand that the information above will be published within the journal article, if accepted, and that failure to comply and/or to accurately and completely report the potential financial conflicts of interest could lead to the following: 1) Prior to publication, article rejection, or 2) Post-publication, sanctions ranging from, but not limited to, issuing a correction, reporting the inaccurate information to the authors' institution, banning authors from submitting work to ASN journals for varying lengths of time, and/or retraction of the published work.

Name: Serge Lemay
Manuscript ID: JASN-2024-000997R1
Manuscript Title: Rac1 suppression by GIT2 and podocyte protection
Date of Completion: December 11, 2024
Disclosure Updated Date: May 12, 2024

ASN American Society of Nephrology JASN Journal of the American Society of Nephrology KIDNEY360 Accessing Our World From Every Angle CJASN Clinical Journal of the American Society of Nephrology

ASN Journal Disclosure Form

As per ASN journal policy, I have disclosed any financial relationships or commitments I have held in the past 36 months as included below. I have listed my Current Employer below to indicate there is a relationship requiring disclosure. If no relationship exists, my Current Employer is not listed.

A. Masztalerz reports the following:
Employer: MUHC

I understand that the information above will be published within the journal article, if accepted, and that failure to comply and/or to accurately and completely report the potential financial conflicts of interest could lead to the following: 1) Prior to publication, article rejection, or 2) Post-publication, sanctions ranging from, but not limited to, issuing a correction, reporting the inaccurate information to the authors' institution, banning authors from submitting work to ASN journals for varying lengths of time, and/or retraction of the published work.

Name: Agnieszka Masztalerz
Manuscript ID: JASN-2024-000997R1
Manuscript Title: Rac1 suppression by GIT2 and podocyte protection
Date of Completion: December 10, 2024
Disclosure Updated Date: December 10, 2024

ASN American Society of Nephrology | JASN Journal of the American Society of Nephrology | KIDNEY360 Accessing Our World From Every Angle | CJASN Clinical Journal of the American Society of Nephrology

ASN Journal Disclosure Form

As per ASN journal policy, I have disclosed any financial relationships or commitments I have held in the past 36 months as included below. I have listed my Current Employer below to indicate there is a relationship requiring disclosure. If no relationship exists, my Current Employer is not listed.

J. Matsuda reports the following:
Employer: Osaka University Graduate School of Medicine

I understand that the information above will be published within the journal article, if accepted, and that failure to comply and/or to accurately and completely report the potential financial conflicts of interest could lead to the following: 1) Prior to publication, article rejection, or 2) Post-publication, sanctions ranging from, but not limited to, issuing a correction, reporting the inaccurate information to the authors' institution, banning authors from submitting work to ASN journals for varying lengths of time, and/or retraction of the published work.

Name: Jun Matsuda
Manuscript ID: JASN-2024-000997
Manuscript Title: Rac1 suppression by GIT2 and podocyte protection
Date of Completion: December 6, 2024
Disclosure Updated Date: December 6, 2024

ASN
American Society of Nephrology
JASN
Journal of the American Society of Nephrology
KIDNEY360
Accessing Our World From Every Angle
CJASN
Clinical Journal of the American Society of Nephrology

ASN Journal Disclosure Form

As per ASN journal policy, I have disclosed any financial relationships or commitments I have held in the past 36 months as included below. I have listed my Current Employer below to indicate there is a relationship requiring disclosure. If no relationship exists, my Current Employer is not listed.

N. Shimada reports the following:
Employer: Osaka University Graduate School of Medicine

I understand that the information above will be published within the journal article, if accepted, and that failure to comply and/or to accurately and completely report the potential financial conflicts of interest could lead to the following: 1) Prior to publication, article rejection, or 2) Post-publication, sanctions ranging from, but not limited to, issuing a correction, reporting the inaccurate information to the authors' institution, banning authors from submitting work to ASN journals for varying lengths of time, and/or retraction of the published work.

Name: Naoyuki Shimada
Manuscript ID: JASN-2024-000997R1
Manuscript Title: Rac1 supression by GIT2 and podocyte protection
Date of Completion: December 8, 2024
Disclosure Updated Date: May 12, 2024

ASN American Society of Nephrology | JASN Journal of the American Society of Nephrology | KIDNEY360 Accessing Our World From Every Angle | CJASN Clinical Journal of the American Society of Nephrology

ASN Journal Disclosure Form

As per ASN journal policy, I have disclosed any financial relationships or commitments I have held in the past 36 months as included below. I have listed my Current Employer below to indicate there is a relationship requiring disclosure. If no relationship exists, my Current Employer is not listed.

T. Takano reports the following:
Consultancy: Otsuka-Canada; GSK-Canada; and Honoraria: Kyowa Kirin, Chugai Pharma.

I understand that the information above will be published within the journal article, if accepted, and that failure to comply and/or to accurately and completely report the potential financial conflicts of interest could lead to the following: 1) Prior to publication, article rejection, or 2) Post-publication, sanctions ranging from, but not limited to, issuing a correction, reporting the inaccurate information to the authors' institution, banning authors from submitting work to ASN journals for varying lengths of time, and/or retraction of the published work.

Name: Tomoko Takano
Manuscript ID: JASN-2024-000997R1
Manuscript Title: Rac1 suppression by GIT2 and podocyte protection
Date of Completion: December 11, 2024
Disclosure Updated Date: May 11, 2024

ASN
American Society of Nephrology
JASN
Journal of the American Society of Nephrology
KIDNEY360
Accessing Our World From Every Angle
CJASN
Clinical Journal of the American Society of Nephrology

ASN Journal Disclosure Form

As per ASN journal policy, I have disclosed any financial relationships or commitments I have held in the past 36 months as included below. I have listed my Current Employer below to indicate there is a relationship requiring disclosure. If no relationship exists, my Current Employer is not listed.

M. Tokuchi has nothing to disclose.

I understand that the information above will be published within the journal article, if accepted, and that failure to comply and/or to accurately and completely report the potential financial conflicts of interest could lead to the following: 1) Prior to publication, article rejection, or 2) Post-publication, sanctions ranging from, but not limited to, issuing a correction, reporting the inaccurate information to the authors' institution, banning authors from submitting work to ASN journals for varying lengths of time, and/or retraction of the published work.

Name: Maho Tokuchi
Manuscript ID: JASN-2024-000997R1
Manuscript Title: Rac1 suppression by GIT2 and podocyte protection.
Date of Completion: December 8, 2024
Disclosure Updated Date: December 8, 2024
