# Supplementary material for: Rac1 Suppression by the Focal Adhesion Protein GIT ArfGAP2 and Podocyte Protection
Source: J Am Soc Nephrol. 2025 Feb 28;36(6):1088–104. doi: 10.1681/ASN.0000000614 (PMC12147964; doi:10.1681/ASN.0000000614)
Supplement: SUPPLEMENTARY MATERIAL [file jasn-36-1088-s003.pdf]

Supplemental Material Table of Contents

Supplemental Methods.

Supplemental Figure 1. Systemic *Git2* deletion exacerbates proteinuria and podocyte foot process effacement after LPS-induced injury in mice.

Supplemental Figure 2. Generation of podocyte-specific *Git2* deficient mice.

Supplemental Figure 3. Heart weight and serum electrolytes are comparable between podocyte-specific *Git2* deficient and control mice in salt-sensitive hypertension model.

Supplemental Figure 4. Gene silencing of *Git2* using another probe causes analogous phenotype, eliminating possible off-targeting effects.

Supplemental Figure 5. Pharmacological inhibition of Rac1 ameliorates accelerated focal adhesion turnover in GIT2 knockdown podocytes.

Supplemental Figure 6. The effect of GIT2 on its paralog GIT ArfGAP1 (GIT1) expression is minimal.

Supplemental Movie 1. Accelerated focal adhesion turnover in GIT2 knockdown podocytes.

Supplemental Movie 2. Pharmacological inhibition of Rac1 ameliorates accelerated focal adhesion turnover in GIT2 knockdown podocytes.

Supplemental Movie 3. Accelerated cell motility in GIT2 knockdown podocytes.

Supplemental Movie 4. Delayed localization of PTP1B to the ruffles in GIT2 knockdown podocytes.

Supplemental Movie 5. GIT2 reconstitution cancelled accelerated focal adhesion turnover in

podocytes with GIT2 knockdown.

Supplemental Methods

Reagents and Antibodies

The antibodies and reagents used are as follows: antibodies for GIT2 (Invitrogen, PA5-78301 for immunoblotting using mouse samples and immunofluorescence staining; Bethyl Laboratories, A302-103A for immunoblotting using human samples; Bethyl Laboratories, A302-102A for immunoprecipitation), tubulin (Sigma-Aldrich, T5168), WT1 (Millipore, 05-753), Rac1 (BD Biosciences, BD610651), phospho-p21 activated kinase (PAK) 1/2/3 (Cell Signaling Technologies [CST], 2606), PAK1/2/3 (CST, 2604), paxillin (Sigma-Aldrich, 05-417), p130 Crk-associated substrate (Cas) (CST, 13846), pY410 p130Cas (CST, 4011), pY118 paxillin (Thermo Fisher Scientific, 44-722G), focal adhesion kinase (FAK) (BD Biosciences, BD610088), pY397 FAK (CST, 8556), Src (CST, 2110), non-pY527 Src (CST, 2107), PTP-PEST (CST, 14735), PTP1B (Santa Cruz Biotechnology, sc-133259), phospho-p44/42 ERK (CST, 9106), p44/42 ERK (CST, 9102), GIT1 (Santa Cruz Biotechnology, sc-398637), lipopolysaccharide (LPS) (Sigma-Aldrich, L2630), Alexa Fluor 647 phalloidin (CST, 8940), deoxicorticostreone acetate (DOCA) (Innovative Research of America, M-121), sodium chloride (Wako, 191-01665), laminin-521 (Corning, 354221), NSC23766 (Fujifilm, 512-38581 for cells and Selleck, S8031 for mice), blebbistatin (Fujifilm, 021-17041), epidermal growth factor (EGF) (Roche, 11376454001), PTP1B inhibitor (Sigma-Aldrich, 539749).

Generation of *Git2* Deficient Mice

Systemic *Git2* knockout (GIT2$^{DEL/DEL}$) and *Git2* floxed mice were established by CLICK at the

Institute of Experimental Animal Sciences of Graduate School of Medicine, the University of Osaka as previously reported.[1] Briefly, 75 ng/µl Cas9 protein (Integrated DNA Technologies [IDT]), two gRNAs (5'-gaccgctctgctaactaggt-3' and 5'-gtggggaacacaattatgtt-3', 20 ng/µl each) (IDT) and 50 ng/µl long single-stranded DNA (lssDNA) (Thermo Fisher Scientifc) were microinjected into pronuclear-stage embryos. Precise sequences of lssDNA which contains exon 2 of *Git2* gene flanked by *loxP* sites were as follows. 5'-atttaccttagctactgttacgcactgtgtgtgtgtcaggatcacacagctctgttattctcaaaaagggactcgacgcggtcatccagatggaggtcagcatagagtgcttgcctagcccagacaaggcccctggctcagtctccagcactggaagcaaacagcggagcaaccacccctgctccctcaatctgctgctctaaccaacatcccctgagagcacgagtctgtggcgtgcacccacatccaggctgcagactggcctgcacaggaaggtgactgctgtgtgcagctgccaaccataacttcgtataatgtatgctatacgaagttattagttagcagagcggtcagtccgggtgggggatcatcagcctcctaggctgaggagcagataaaacaacaaaaatgtgctctgtttttcttaaaaaccaaacatcactcatcttttgccatcctgtacacagcagccaattggtgtccttgccactgaggggttgttcttttccagatccttcctgggcgtcagtaaataggggaactttcatatgtgacgaatgttgcagtgtccatcggagcctggggcgccatatctcccaagtgaggcatcttaaacacacagcatggcctccaaccctgctccaggtaaaccataggaagagttctcataattggaaaattatattttagtgattgttgcttaacaactaaaatactgcctactatacatcttaaatcttataaaagtttactctctatgtatagtataaagtgctgccctgtgttgaagtcattgtctgtcctttctttgtttacctaacataacttcgtataatgtatgctatacgaagttatataattgtgttccccacattcagttatacatagacagatatatcaatagattgtgtcg-3'.

Direct sequencing analysis was performed, and mice carrying heterozygous large deletion alleles ($GIT2^{DEL/+}$) and floxed alleles ($GIT2^{flox/+}$) were used for further breeding to generate $GIT2^{DEL/DEL}$ and $GIT2^{flox/flox}$ mice, respectively.

Genotyping

To determine the genotype, mice DNA was extracted from tails by KAPA Express Extract Kit (Kapa Biosystems, KK7102) and subjected to PCR. The genotyping primer sequences used for *Cre* were described previously,[2] and those for *Git2* were as follows:

F 5'-ttagcacctgggtctggact-3'

R1 5'-cgatggacactgcaacattc-3'

R2 5'-gcacggttgcaattaccttt-3'

F and R1 primers were used to detect floxed sequence, and F and R2 primers were used to detect large deletion.

Isolation of Glomeruli

The isolation of glomeruli was performed as previously described.[2,3]

Immunoblot Analysis

Cells and mice tissues were lysed with ice-cold lysis buffer (CST, 9803) and protease inhibitor cocktail (Roche, 11697498001). Protein concentrations were measured and quantitative densitometry was performed as previously described.[2] Images were obtained using ChemiDoc Touch MP (Biorad) according to the manufacturer's instructions.

In Situ Hybridization

In situ hybridization assay was performed using RNAscope (Advanced Cell Diagnostics, Inc.) with the target probe for mouse *Git2* (Mm-Git2-C1 probe, #1317731-C1), *Wt1* (Mm-Wt1-C2, #432711-

C2), *Pdgfr* (Mm-PDGFRb-C3, #411381-C3) and *Pecam1* (Mm-PECAM1-C4, #316721-C4) mRNA according to the manufacturer's instructions.

LPS-induced Proteinuria Model

Male 8-9 week-old mice were intraperitoneally injected with LPS as described previously.[2] NSC23766 (10mg/kg body weight/day) was subcutaneously infused using an osmotic pump (Alzet, 2002) starting from 4 days before LPS injection. Urine was collected at 0, 6, 24 and 48 hours after treatment and analyzed for ACR. Kidneys were harvested at 48 hours and subjected to electron microscopic analysis.

Salt-sensitive Hypertension Model

Male 8-9 week-old mice were uninephrectomized and subcutaneously implanted continuous 21-day release pellets containing 50mg DOCA. Simultaneously, subcutaneous infusion of NSC23766 (10mg/kg body weight/day) was started using the same pump as above. Drinking water was replaced with 1% sodium chloride as previously reported.[4] Urine was collected at 0, 7-8 and 13-14 days after treatment, and analyzed for ACR. Blood pressure was measured by tail cuff system (Softron, Tokyo, Japan) before and at 14 days after treatment. Kidneys were harvested at 14 days and subjected to electron and light microscopic analysis.

Electron Microscopic Analysis

Kidneys were fixed with 2% glutaraldehyde (TAAB Laboratories, G013). Micrographs of 4 to 10 tufts per glomerulus were randomly captured using a 120kV Hitachi H-7650 transmission electron

microscope (Hitachi, Tokyo, Japan). Analysis for foot process width has been described previously.[5]

Biochemical Measurements

The urinary albumin and creatinine levels were measured as described previously.[2] Serum levels of sodium, potassium, chloride and urea nitrogen were measured at Oriental Yeast (Tokyo, Japan).

Light Microscopic Analysis

Kidneys were fixed in 4% paraformaldehyde. Embedding in paraffin and periodic acid Schiff staining were performed by Applied Medical Research Laboratory (Osaka, Japan). Tubulointerstitial damage was quantified by scoring as described previously.[6]

Plasmid Constructs

mRFP-tagged paxillin, EGF receptor (EGFR), pEBG-tagged PTP1B and PTP-PEST, and GFP-tagged PTP1B have been described previously.[7-9]

To generate lentiviral GIT2 construct, cDNA of human GIT2 was amplified from pcDNA3.1(+)-C-eGFP GIT2 vector (GenScript, OHu19439C) using primers below, digested by SwaI (CST, R0604) and NotI (CST, R0189), and ligated into pLVSIN-EF1a Hyg vector (Takara, 6185) using T4 DNA Ligase (CST, M0202) according to the manufacturer's instructions. The product was verified by Sanger sequencing.

F 5'-ggaaggatttaaatatgtcgaaacggctccggag-3'

R 5'-aaggaaaaaagcggccgctcagttgttgttctctttggtggtga-3'

Lentiviral Transduction

Podocytes with GIT2 knockdown (KD) and their controls (CTRL) were established using lentiviral shRNA (Vector Builder, VB900124-8420kmf [KD#1] and VB010000-0007mbh [CTRL#1]; Sigma-Aldrich, TRCN0000008407 [KD#2], SHC001 [CTRL#2] and SHC002 [CTRL#3]). HEK293T cells (Clontech, 632180) were transiently transfected using the lentiviral packaging system (Sigma-Aldrich, SHP001) according to the manufacturer's instructions. Virus-containing supernatants were added to podocytes under permissive conditions for 16 hours. Blasticidin (Fujifilm, 026-18711)-resistant (KD#1 and CTRL#1) or puromycin (Sigma-Aldrich, P9620)-resistant (KD#2, CTRL#2 and CTRL#3) cells were pooled for further experiments.

GIT2-overexpressing podocytes were established by the same lentiviral approach as above using human GIT2 construct except for the use of hygromycin (Fujifilm, 084-07681) for the selection. Cells transfected with empty plasmids were used for the controls.

Cell Spreading Assay

Cells were seeded onto coverslips coated with 0.25 μg/cm^2 laminin-521, and fixed with 4% paraformaldehyde at 2hours after plating followed by phalloidin staining.

Immunofluorescence Analysis

Immunofluorescence staining was performed as described previously.[10] Images were obtained using a Zeiss LSM880 laser scanning confocal microscope (Zeiss, Jena, Germany) with the Zeiss Plan Apochromat 63x 1.40 Oil DIC objective. All imaging parameters were maintained constant throughout image acquisition of all samples. To evaluate three-dimensional localization, image

sequences captured by Z-stack mode were transferred to and reconstructed by Imaris software (Oxford instruments). Measurement of cell area and quantification of focal adhesion complex were performed using ImageJ software as described previously.[10,11] The number of paxillin-positive particles between 0.1-8.0 μm^2 was counted. Focal adhesions were classified into four groups: 0.1-0.5 μm^2, 0.5-1.0 μm^2, 1.0-3.0 μm^2 and 3.0-8.0 μm^2.

Rho-GTPase Pull-down Assay

Preparation for GST-CRIB beads was performed as described previously.[2] After washing once with ice-cold PBS, cells were lysed with the lysis buffer (above) and mixed with the beads for 1.5 hour at 4^oC. Beads were washed three times, and proteins were eluted from beads into SDS loading buffer.

Co-immunoprecipitation Assay

Cell lysates were incubated with the anti-GIT2 antibody overnight at 4^oC, and then with protein A agarose beads (Santa Cruz, sc-2001) for 1.5 hour at 4^oC. Beads were washed three times, and proteins were eluted from the beads into SDS loading buffer. Normal rabbit IgG (CST, 2729) was used as a negative control.

Time-lapse Live Cell Imaging

For time-lapse live cell imaging, cells were transfected with mRFP-paxillin and/or GFP-PTP1B using Lipofectamine2000 (Invitrogen). Cells were then seeded at ～30% confluence on glass-bottomed dishes coated with 0.25 $\mu g/cm^2$ laminin-521. At 24-48 hours after differentiation, live cell images were captured every 2 minutes using a ZEISS LSM880 confocal laser scanning microscope. During

microscopy cells were maintained at 37˚C and under 5% CO_2 condition. Images were taken under the same laser power and pinhole diameter conditions, and significant photobleaching was not detected. RFP (paxillin) at the leading edge of the cells was focused and used for analysis. Focal adhesion turnover was quantified as previously reported.[7] Briefly, focal adhesion lifetimes were determined by measuring the time elapsed between the first and last frame in which each RFP-positive focal adhesion appeared. The average fluorescent intensity of RFP in individual adhesions was measured over time using ZEISS ZEN software, and the background intensity was subtracted from the values. For rate constant measurements, periods of assembly (increasing fluorescence intensity) and disassembly (decreasing fluorescence intensity) of focal adhesions were plotted up to 10 minutes. Semi-logarithmic plots of fluorescence intensities as a function of time were generated using the formulas, Ln([I]/[I0]) for assembly and Ln([I0]/[I]) for disassembly. I0 and I are fluorescence intensity at the initial and various time points, respectively. Linear regression trend lines fitted to the plots were then drawn to determine apparent rate constants of assembly and disassembly.

Wound Healing Assay

Cells were seeded onto a 96-well IncuCyte® ImageLock microplate (Sartorius, Göttingen, Germany) coated with 0.25 μg/cm^2 laminin-521 at a density of 18,000-20,000 cells per well. After cells reached a confluent state, a scratch wound was made by the IncuCyte 96-well Wound Maker (Sartorius). Cells were then kept under non-permissive conditions up to 20 hours. The wound confluence, defined as the percentage of cell confluence within the wound region, was analyzed over time by IncuCyte

Analysis Software (Sartorius).

Substrate Trapping Assay

HEK293 cells were transfected with indicated pEBG-tagged and EGFR plasmids using lipofectamine2000. After 100 ng/ml EGF stimulation for overnight, cells were lysed and the same amounts of lysates were pulled down with glutathione sepharose beads (Cytiva, 17-5279-01).

References

1. Miyasaka Y, Uno Y, Yoshimi K, et al.: CLICK: one-step generation of conditional knockout mice. *BMC Genomics* 19: 318, 2018. doi: 10.1186/s12864-018-4713-y
2. Matsuda J, Maier M, Aoudjit L, Baldwin C, Takano T: ARHGEF7 (beta-PIX) Is Required for the Maintenance of Podocyte Architecture and Glomerular Function. *J Am Soc Nephrol* 31: 996-1008, 2020. doi: 10.1681/ASN.2019090982
3. Takemoto M, Asker N, Gerhardt H, et al.: A new method for large scale isolation of kidney glomeruli from mice. . *Am J Pathol* 161: 799-805, 2002. doi,
4. Tavasoli M, Li L, Al-Momany A, et al.: The chloride intracellular channel 5A stimulates podocyte Rac1, protecting against hypertension-induced glomerular injury. *Kidney Int* 89: 833-847, 2016. doi: 10.1016/j.kint.2016.01.001
5. Kumagai T, Baldwin C, Aoudjit L, et al.: Protein tyrosine phosphatase 1B inhibition protects against podocyte injury and proteinuria. *Am J Pathol* 184: 2211-2224, 2014. doi: 10.1016/j.ajpath.2014.05.005
6. Zhu L, Jiang R, Aoudjit L, Jones N, Takano T: Activation of RhoA in podocytes induces focal segmental glomerulosclerosis. *J Am Soc Nephrol* 22: 1621-1630, 2011. doi: 10.1681/ASN.2010111146
7. Burdisso JE, Gonzalez A, Arregui CO: PTP1B promotes focal complex maturation, lamellar persistence and directional migration. *J Cell Sci* 126: 1820-1831, 2013. doi: 10.1242/jcs.118828
8. Aoudjit L, Jiang R, Lee TH, New LA, Jones N, Takano T: Podocyte Protein, Nephrin, Is a Substrate of Protein Tyrosine Phosphatase 1B. *J Signal Transduct* 2011: 376543, 2011. doi: 10.1155/2011/376543
9. Baldwin C, Chen ZW, Bedirian A, et al.: Upregulation of EphA2 during in vivo and in vitro renal ischemia-reperfusion injury: role of Src kinases. *Am J Physiol Renal Physiol* 291: F960-971, 2006. doi: 10.1152/ajprenal.00020.2006
10. Matsuda J, Greenberg D, Ibrahim S, et al.: CdGAP maintains podocyte function and modulates focal adhesions in a Src kinase-dependent manner. *Sci Rep* 12: 18657, 2022. doi: 10.1038/s41598-022-21634-1

11. Kachurina N, Chung CF, Benderoff E, et al.: Novel unbiased assay for circulating podocyte-toxic factors associated with recurrent focal segmental glomerulosclerosis. *Am J Physiol Renal Physiol* 310: F1148-1156, 2016. doi: 10.1152/ajprenal.00349.2015

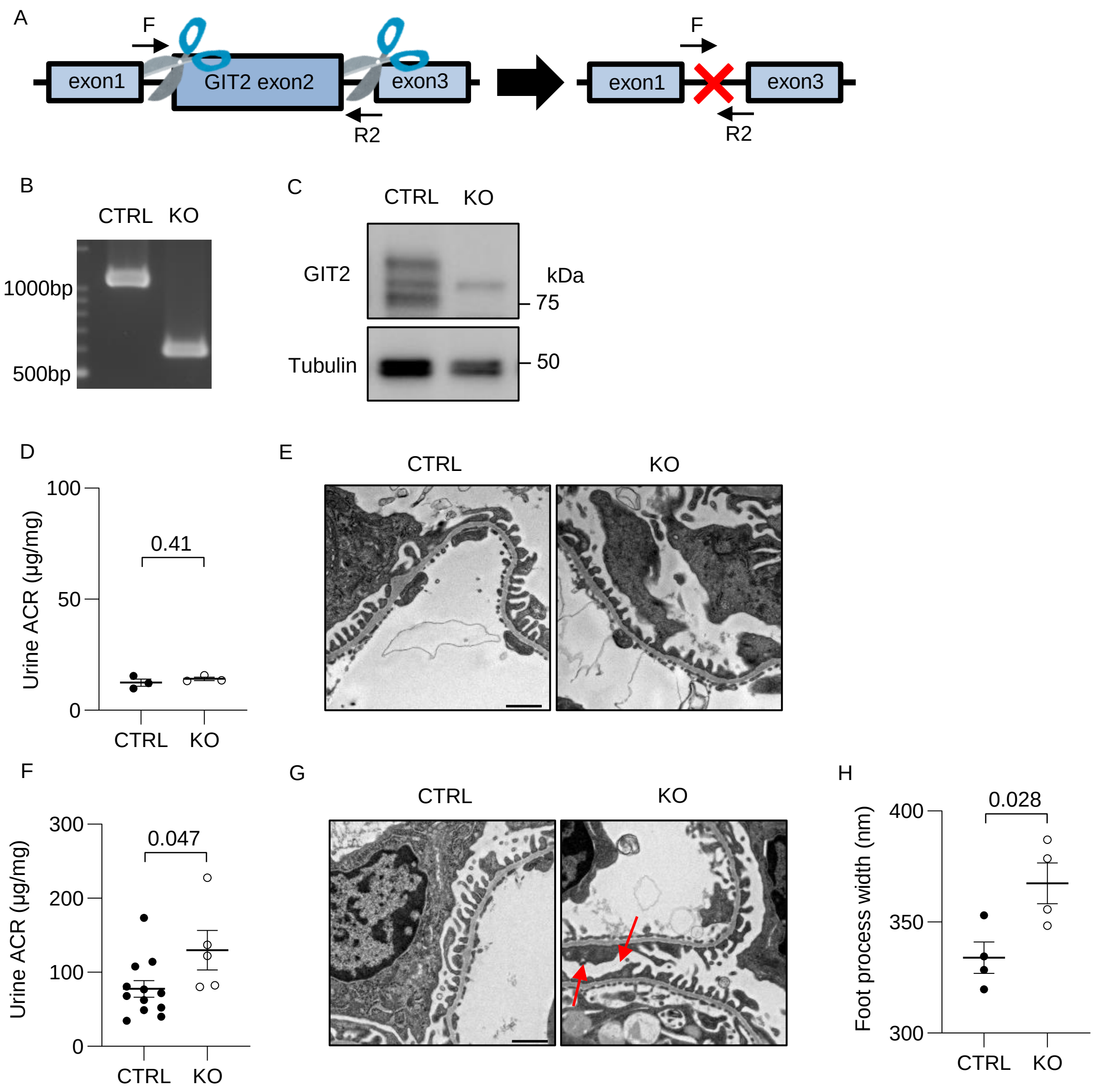

Supplemental Figure 1. Systemic *Git2* deletion exacerbates proteinuria and podocyte foot process effacement after LPS-induced injury in mice.
(**A**) Design of CRISPR/Cas9 system. The exon2 of *Git2* gene was excised to generate systemic *Git2* deficient (knockout [KO]) mice. Primers used for genome PCR in (**B**) were shown (arrows). (**B**) Representative PCR analysis of the extracted DNA obtained from tails of systemic KO and control (CTRL) mice. PCR product bands of non-excised sequence (1095 bp) in control mice and excised sequence in KO mice are shown. (**C**) Representative immunoblots for GIT2 (75 and 85 kDa) and tubulin using lysates of isolated glomeruli from KO and control mice. Note that a faint band (just above 75 kDa) observed in the lane of KO mice is non-specific. (**D** and **E**) Quantification of urine albumin to creatinine ratio (ACR) (**D**) and representative images of transmission electron microscopy showing the glomeruli (**E**) from 6-month-old KO and control mice. (**F**) Quantification of urine ACR from 8-9-week-old KO and control mice after 48 hours of LPS injection. (**G**) Representative images of transmission electron microscopy showing the glomeruli after 48 hours of LPS injection. Red arrows show podocyte foot process effacement in KO mice. GBM thickness and fenestra of the glomerular endothelial cells were comparable between groups. (**H**) Quantification of podocyte foot process width in (**G**). n = 3 (**D**); 5 or 12 (**F**); 4 (**H**) in each group. Bars: 1 µm (**E** and **G**).

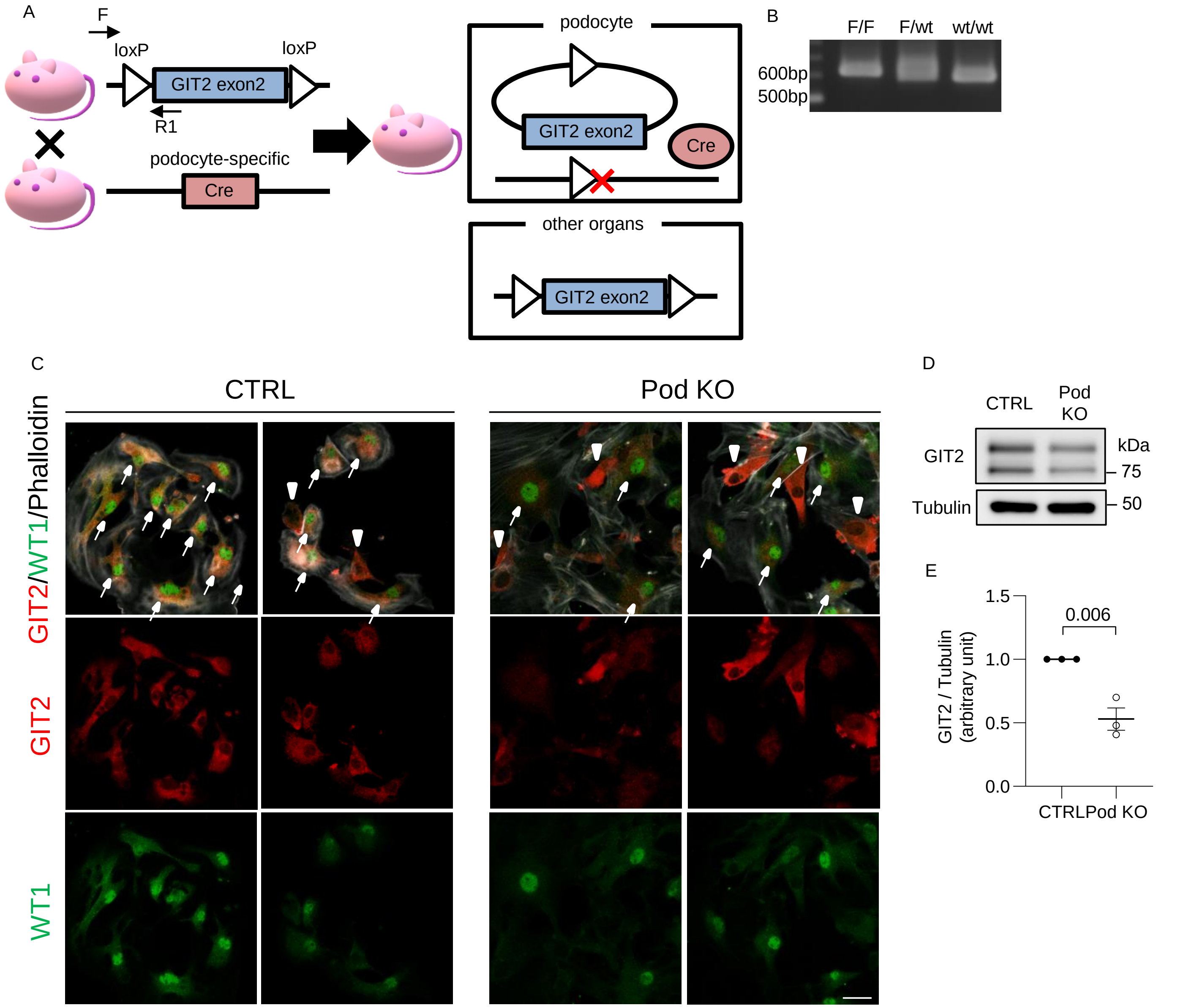

Supplemental Figure 2. Generation of podocyte-specific *Git2* deficient mice.
(**A**) Using CRISPR/Cas9 system, *loxP* sequences were inserted before and after the exon2 of *Git2* gene. Floxed mice were crossed with *Nphs2 Cre* mice to generate podocyte-specific *Git2* knockout mice. Primers used for genome PCR in (**B**) were shown (arrows). (**B**) Representative PCR analysis of the extracted DNA obtained from tails of wild-type (wt/wt), heterozygous *Git2*-floxed (F/wt) and homozygous *Git2*-floxed (F/F) mice. PCR product bands of sequences with *loxP* (floxed; 625 bp) or without *loxP* (wt; 591 bp) sequences are shown. Cre-positive homozygous *Git2*-floxed mice were regarded as podocyte-specific knockout (Pod KO). (**C**) Representative images of immunofluorescence staining using primary outgrowth of isolated glomeruli. GIT2 was detected in WT1-expressing podocytes from control (CTRL) mice, but was absent in those from Pod KO mice (arrows). WT1-negative cells (non-podocyte glomerular cells) from Pod KO mice showed distinct staining of GIT2 (arrowheads), indicating podocyte-specific GIT2 deletion. (**D**) Representative immunoblots for GIT2 and tubulin using lysates of isolated glomeruli from Pod KO mice and their age-matched controls. (**E**) Densitometric quantification in (**D**). n = 3 (**E**) in each group. Bar: 20 µm (**C**).

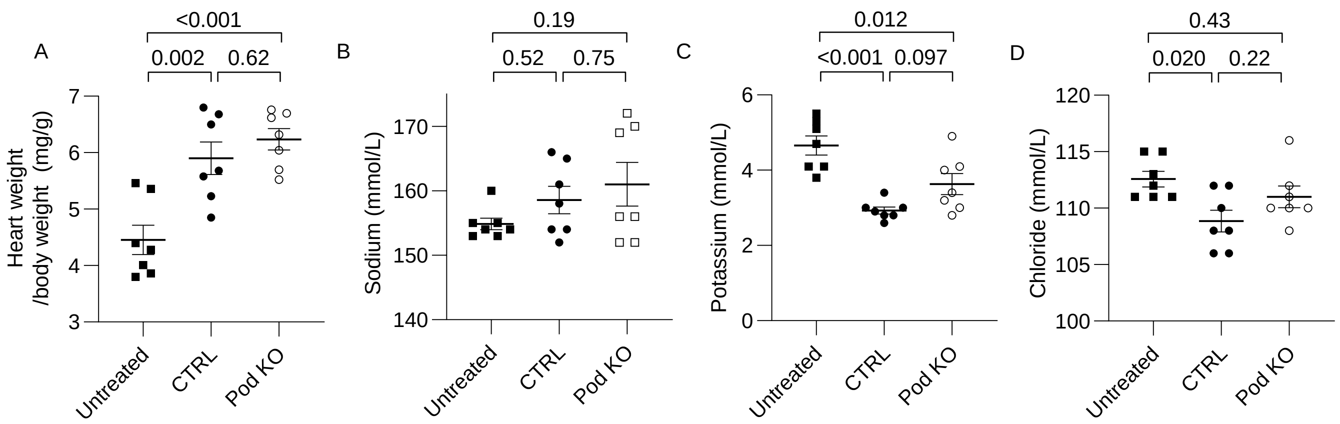

Supplemental Figure 3. Heart weight and serum electrolytes are comparable between podocyte-specific *Git2* deficient and control mice in salt-sensitive hypertension model.
The ratio of heart weight to body weight (**A**) and serum levels of sodium (**B**), potassium (**C**) and chloride (**D**) from uninephrectomy-DOCA/salt-treated podocyte-specific *Git2* deficient (Pod KO) and control (CTRL) mice at day 14 are shown. The levels from untreated mice are also shown as a reference. n = 7 in each group.

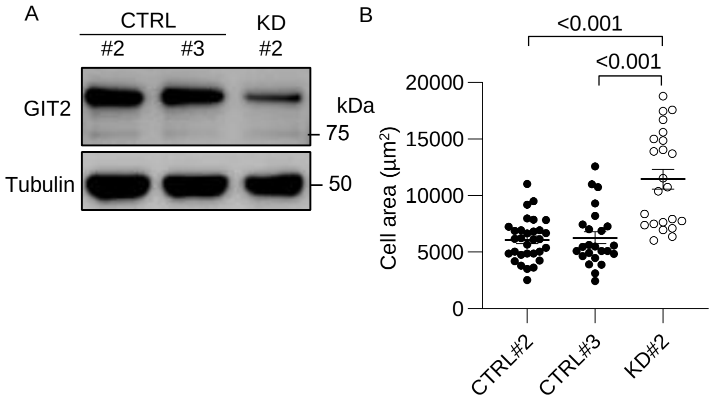

Supplemental Figure 4. Gene silencing of *GIT2* using another probe causes analogous phenotype, eliminating possible off-targeting effects.

(**A**) Representative immunoblots for GIT2 and tubulin of cultured human podocytes with GIT2 knockdown (KD#2) and controls (CTRL#2 and #3). (**B**) Quantification of the cell area of differentiated GIT2 knockdown and control podocytes. n = 23 to 31 in each group (**B**).

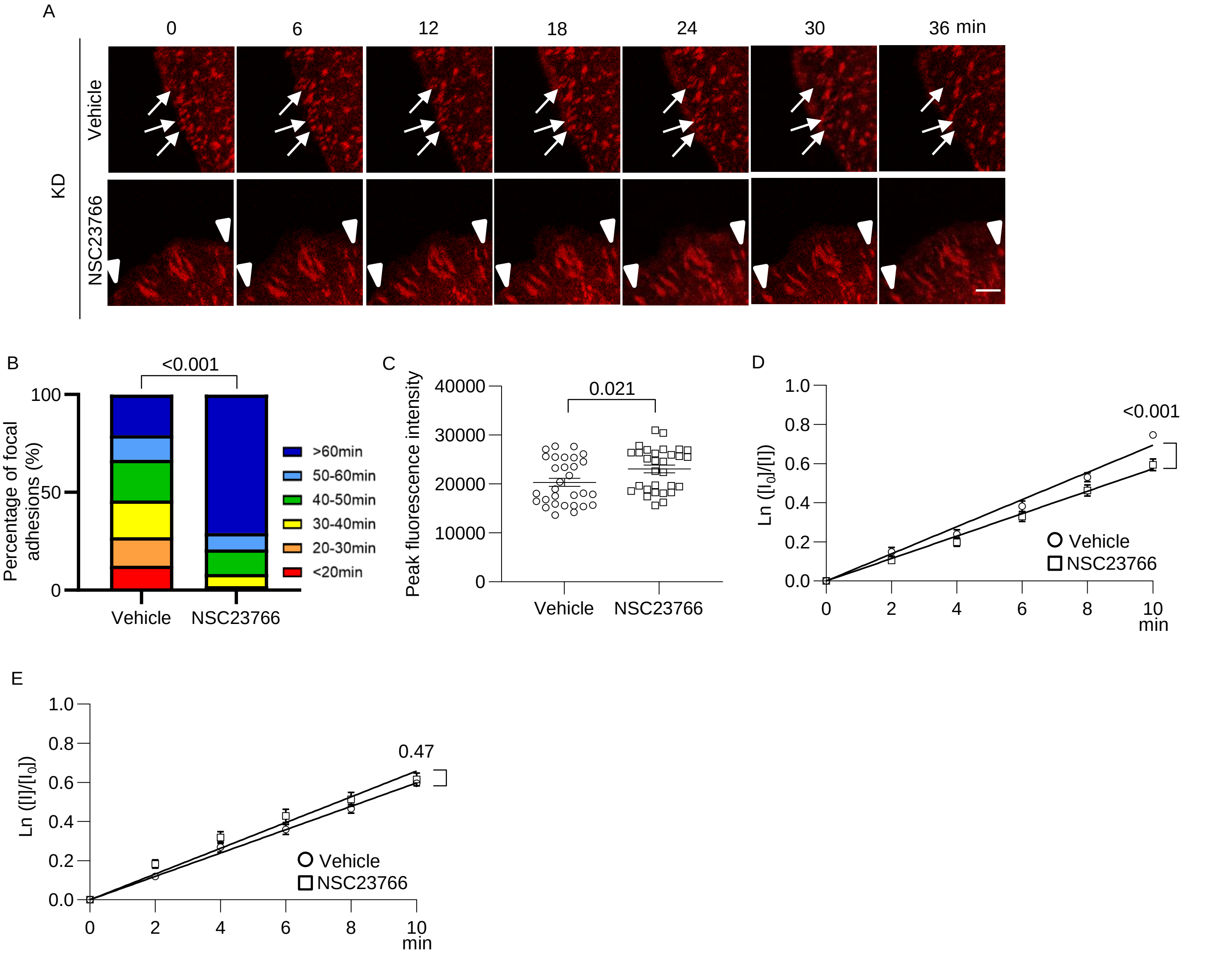

Supplemental Figure 5. Pharmacological inhibition of Rac1 ameliorates accelerated focal adhesion turnover in GIT2 knockdown podocytes.

(**A**) Representative images of the time-lapse confocal microscopy. GIT2 knockdown (KD) podocytes transiently transfected mRFP-tagged paxillin were treated with either 25μM NSC23766 or vehicle for 2 days. Most focal adhesions labelled by mRFP-paxillin in vehicle-treated GIT2 knockdown podocytes are short-lived (arrows) while matured in NSC23766-treated knockdown cells (arrowheads). (**B**) Distribution of focal adhesion lifetime in (**A**) is shown. (**C**) Quantification of the peak focal adhesion intensity in (**A**). (**D** and **E**) Quantification of focal adhesion disassembly (**D**) and assembly (**E**) rates in (**A**) are shown. n = 48 focal adhesions in 4 or 5 cells (**B**); 30 focal adhesions in 5 cells (**C**); 19 or 20 focal adhesions in 3 or 4 cells (**D** and **E**) in each group. Bar: 5 μm (**A**).

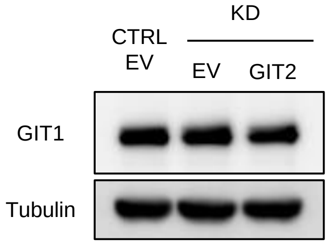

Supplemental Figure 6. The effect of GIT2 on its paralog GIT ArfGAP1 (GIT1) expression is minimal.
Either human GIT2 or empty vector (EV) was transfected into podocytes. Representative immunoblots for GIT1 and tubulin of cultured control (CTRL), GIT2 knockdown (KD) and GIT2-overexpressiong podocytes. The protein abundance of GIT1 is similar among the groups.
